# Development and evaluation of machine learning-based prediction-modelling for initial vancomycin serum concentrations in septic ICU patients using clinical health record data

**DOI:** 10.1186/s40635-026-00973-7

**Published:** 2026-09-23

**Authors:** Robin Grugel, Britta Westhus, Hartmuth Nowak, Michael Adamzik, Tim Rahmel, Martin Eisenacher

**Affiliations:** 1 Medical Faculty, Medizinisches Proteom-Center (MPC), Ruhr University Bochum, 44801 Bochum, Germany; 2 Medical Faculty, Center for Protein Diagnostics (ProDi), Ruhr University Bochum, 44801 Bochum, Germany; 3 Ruhr University Bochum, Knappschaft Kliniken University Hospital Bochum, Department of Anesthesiology, Intensive Care Medicine and Pain Therapy, 44892 Bochum, Germany; 4 Ruhr University Bochum, Knappschaft Kliniken University Hospital Bochum, Department of Anesthesiology, Intensive Care Medicine and Pain Therapy, Center for Artificial Intelligence, Medical Informatics and Data Science, 44892 Bochum, Germany; 5 Medical Faculty, Core Unit Bioinformatics (CUBiMed.RUB), Ruhr University Bochum, Bochum, Germany; 6 Medical Faculty, BioInfra.Prot, Deutsches Netzwerk für Bioinformatik-Infrastruktur (de.NBI), Ruhr University Bochum, Bochum, Germany

**Keywords:** Vancomycin, Sepsis, Machine learning, Prediction

## Abstract

**Background:**

Sepsis remains a life-threatening condition with highly heterogeneous and dynamic pathophysiology, limiting the effectiveness of uniform therapeutic strategies. Beyond timely source control, antimicrobial therapy represents the only causal treatment option. Vancomycin is widely used for treatment of Gram-positive infections; however, optimal dosing in septic patients is challenging due to pronounced pharmacokinetic variability and substantial interindividual heterogeneity. Underdosing may promote antimicrobial resistance, whereas overdosing increases the risk of toxicity. This study aimed to develop and validate a machine learning-based prediction model to support individualized vancomycin dosing using routinely available clinical data.

**Methods:**

This single-center retrospective study included adult sepsis patients admitted to the intensive care unit, using routinely collected data from the hospital’s electronic medical records. Patients were eligible if they received a vancomycin loading dose followed by continuous infusion and had at least one measured serum concentration. Three machine learning models–elastic net regression, random forest, and XGBoost–were developed to predict the initial vancomycin serum concentration. To minimize bias and enhance generalizability, model training, hyperparameter tuning, and performance evaluation were conducted using a stratified nested cross-validation approach. Model performance was compared with seven commonly used population pharmacokinetic models.

**Results:**

The developed best performing elastic net model achieved a notable improvement with an average RMSE of 6.19, compared to 7.83 for the best pharmacokinetic model highlighting the potential of early and individualized dosing supported by a machine learning model. Final model analysis revealed that noradrenaline administration, together with classical pharmacokinetic parameters including body weight, serum creatinine, and the presence of chronic kidney disease, significantly influenced predictive performance.

**Conclusions:**

This machine learning-based approach for predicting initial vancomycin serum concentrations outperforms conventional PK models and enables more precise, individualized dosing prior to the availability of therapeutic drug monitoring results. By integrating key clinical variables, the model facilitates data-driven decision-making in sepsis care and underscores the potential of machine learning to advance personalized antimicrobial therapy.

## Background

Sepsis is defined as a life-threatening organ dysfunction caused by a dysregulated host response to infection [1]. Despite major advances in critical care, sepsis-associated mortality rates remain high [2]. This is due to the complex pathophysiology of sepsis, driven by many interacting biological and clinical factors, resulting in heterogeneity, multidimensionality, and non-linear disease dynamics [3, 4]. As a result, the development of uniform causal therapies has been limited, leaving source control and antimicrobial treatment as the only interventions that directly targeted the underlying infection [5–7].

Vancomycin remains a cornerstone in the treatment of severe Gram-positive infections, particularly those caused by methicillin-resistant *Staphylococcus aureus* [8, 9]. Its clinical management, however, is challenging. Vancomycin exhibits complex pharmacokinetic (PK) and pharmacodynamic (PD) properties, and drug exposure varies widely among critically ill patients due to dynamic physiological alterations, organ dysfunction, and concurrent therapeutic interventions [10, 11]. Achieving optimal exposure is crucial, as inappropriate dosing may have serious clinical consequences [12]. Underdosing can result in subtherapeutic concentrations, inadequate bacterial eradication, and the emergence of resistant strains such as vancomycin-intermediate S. aureus (VISA). In contrast, overdosing increases the risk of nephrotoxicity, particularly in patients with impaired renal function or concomitant nephrotoxic medications [8, 13]. Both scenarios are associated with poorer clinical outcomes, prolonged intensive care unit (ICU) stay, and increased mortality. Effective vancomycin therapy requires individualized dosing strategies, considering each patient’s condition, including organ function, inflammation, and hemodynamic status, along with any interventions, such as ventilation or dialysis [14, 15].

Therapeutic drug monitoring (TDM) supports dose individualization during ongoing therapy, by enabling dose adjustments based on measured serum concentrations [16]. However, no concentration data are available at the time of the first dose. Accurate initial dosing is particularly critical during early sepsis management, where timely and adequate antibiotic administration is essential, as emphasized in the “1-hour sepsis bundle” [6]. In clinical practice, initial dosing decisions largely rely on population-based estimates that fail to reflect the pronounced interpatient variability observed in septic ICU patients [15, 17]. Moreover, TDM is not universally available, and limited infrastructure or slow turnaround can delay early dose optimization, reinforcing the need for alternative data-driven strategies to individualize dosing from the start of therapy.

Pharmacometric approaches, including population pharmacokinetic (PopPK) models and model-informed precision dosing (MIPD) tools, have been developed to improve individualized dosing [18–20]. In statistical modelling, tools such as TDMx apply Bayesian model averaging or model selection to improve the precision of predictions across multiple PopPK models once TDM data become available [19]. However, the efficacy of these approaches is contingent upon the availability of measured concentrations, which are essential for identifying the most suitable model. As demonstrated by [19], for the first dose–before any TDM data exist–TDMx reverts to a single, manually selected PopPK model, limiting its ability to provide personalized initial dosing [19]. Furthermore, existing PopPK models were generally not developed in septic ICU populations and rely on a restricted set of covariates such as age, body weight, and serum creatinine, which are insufficient to capture the profound inter- and intraindividual variability characteristic of sepsis. The development of new PopPK models using real-world ICU data is challenging, as vancomycin concentration measurements are often sparse, irregular, and influenced by clinical constraints. In this context, machine learning (ML) offers a promising alternative by using routinely collected clinical data to predict drug exposure. Although recent studies [21–26] have applied ML methods to guide vancomycin therapy, the majority of these studies have focused on classification tasks or dose recommendations rather than direct prediction of serum concentrations. However, such prediction can be essential for interpretable and clinically applicable dosing support. To address this gap, the present study aimed to develop and validate an ML-based model to predict initial vancomycin serum concentrations in septic ICU patients using routinely collected electronic health record data as additional important predictors. By estimating expected concentrations before TDM becomes available, this approach reflects real-world clinical decision-making and enables individualized dosing from the start of therapy. Model performance was compared with established PopPK models to evaluate its accuracy and potential to improve personalized antimicrobial treatment in sepsis.

**Fig. 1:**
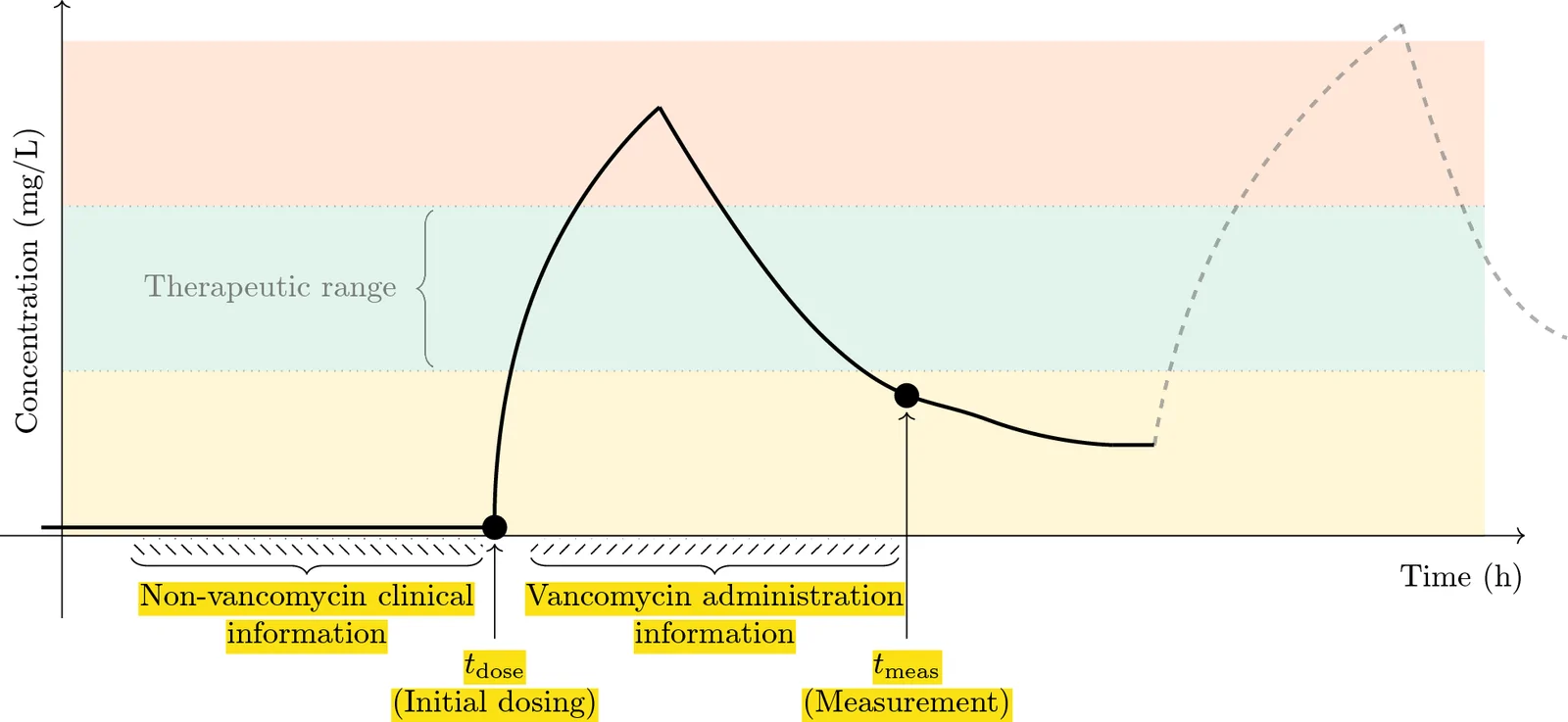
Illustrative example of a time-concentration curve with two intervals contributing different predictive information to the modelling of concentration measurements

## Methods

In all modelling decisions, we aimed to report and document our approach as transparently as possible, based on the TRIPOD+AI recommendations [27] as an update of the original TRIPOD guidelines [28], while applying a strict and statistically more robust evaluation methodology. It is important to emphasize that our modelling strategy serves two purposes: first, to evaluate performance relative to a baseline using appropriate internal validation (nested resampling), and second, to interpret the final selected and estimated model with respect to predictor effects.

### Clinical cohort data

The data were obtained as part of the SepsisDataNet.NRW study from 2018 until 2022, a multi-center registry investigating the clinical and biological characteristics of sepsis and its antibiotic treatment across several hospitals in North Rhine-Westphalia. For the present analysis, we included only patients treated at one of these participating centers, Ruhr-University Bochum, Knappschaft Kliniken University Hospital Bochum, since this site provided a particularly comprehensive set of clinical variables for the patients meeting our inclusion criteria. The study should therefore be regarded as a single-center analysis within the broader SepsisDataNet.NRW registry, whose ethics approval by the committee of Ruhr-University Bochum (registry No. 18–6606-BR) covers all participating centers even though only one center’s data were used here. The resulting dataset encompasses demographic information, comorbidities, vital signs, ventilatory status, medication administration, fluid balance, and clinical scores, all represented as high-resolution time-series data. For model development, patients were included if they had received at least one vancomycin loading and maintenance dose within 12–120 hours prior to the first concentration measurement. This interval also reflects the recommended time frame for adjusting continuous infusion therapy after approximately 24 hours. The final cohort comprised 64 patients meeting these criteria, necessitating careful selection of appropriate machine learning methods for analysis.

### Prediction problem and modelling strategy

The aim of this study was to predict the first vancomycin serum concentration following initial dosing. Because the model is intended to support clinical dosing decisions, it is essential to define which patient information is available at the time of dose selection. Figure 1 illustrates two key time points: (1) the decision time point $t_{\text {dose}}$ at which the initial vancomycin dose is prescribed and (2) the time point $t_{\text {meas}}$ at which the first serum concentration is measured. To reflect this clinical setting and to be able to provide dosing guidance in newly admitted patients, variables available before time point $t_{\text {dose}}$ were considered as predictors that are incorporated in to the model. Only information on the vancomycin treatment^1^ is used between time point $t_{\text {dose}}$ and $t_{\text {meas}}$. Together, these intervals establish a structured framework for selecting and preprocessing the clinical variables used in the ML models.

### Pre-selection of predictor variables

Given the large amount of available information in the electronic health record in this small-sample setting, a preliminary selection of potentially relevant clinical variables was performed. In addition to the core information regarding the administration of vancomycin, including its dosage and the duration of the ICU stay, predictor variables were selected based on clinical plausibility and previously reported associations with vancomycin pharmacodynamics or the characteristics of sepsis. Demographic and anthropometric characteristics included sex [29, 30], age [29, 30], and body weight, height or body mass index (BMI) [31, 32]. Laboratory parameters comprised renal function markers such as glomerular filtration rate (GFR; via Cockcroft-Gault-formula) [29, 31, 33], creatinine [29, 32, 34], urea [30], and changes in renal function [31]; hepatic function indicators including bilirubin, glutamic oxaloacetic transaminase (GOT) [35] and prothrombin time (Quick test) [33, 36]; as well as inflammatory and hematologic markers such as C-reactive protein (CRP) [30], platelet count [34], red blood cell distribution (RDW) [37], procalcitonin (PCT) [38] or Nucleated red blood cells (NRBC) [39]. Intervention-related variables encompassed mechanical ventilation, catecholamine or norepinephrine infusion, and high fluid administration [33], as well as dialysis [40]. Vital parameters included mean arterial pressure (MAP), Horowitz index (PaO_2_/FiO_2_ ratio), and cumulative urine output per day [34]. Additional variables included the SOFA score [31, 34, 41], comorbidities such as pre-existing kidney disease and diabetes mellitus [30]. SOFA scores were determined by the treating clinical team as part of routine ICU documentation and entered into the model as recorded, without being recalculated from their individual components. In sedated or intubated patients, the Glasgow Coma Scale component reflected the patient’s neurological status immediately before sedation was initiated, consistent with standard clinical scoring practice in this setting. Very few predictor pairs showed a relevant correlation, and none were excluded on the basis of collinearity (see correlation matrix in Supplementary material).

### Aggregation of raw data

The aggregation of longitudinal data was based on two distinct time intervals, with the aggregation method varying according to the variable meaning. Demographics and comorbidities, as relatively temporally invariant variables, were included without aggregation. For already cumulative variables and physical characteristics such as body weight and height, only the most recent value prior to $t_{\text {dose}}$ was retained. Vital signs and laboratory measurements were summarized within the 24-hour interval prior to $t_{\text {dose}}$ using the mean. Although additional summary statistics (e.g., minima, maxima, variability measures, temporal slopes, or lagged values of summary statistics for multiple intervals), or more elaborate approaches such as functional data analysis capturing the entire trajectory, were conceptually possible and might further improve predictive performance, they would increase the predictor space (or model complexity) too much for our limited sample size for this modelling task. Thus, the mean was selected as a crude but sufficient representation of these time-varying variables. Although the duration of the aggregation window could in principle be chosen differently, a 24-hour interval was selected because it captures a clinically relevant amount of information given the rapidly evolving nature of sepsis.

**Fig. 2:**
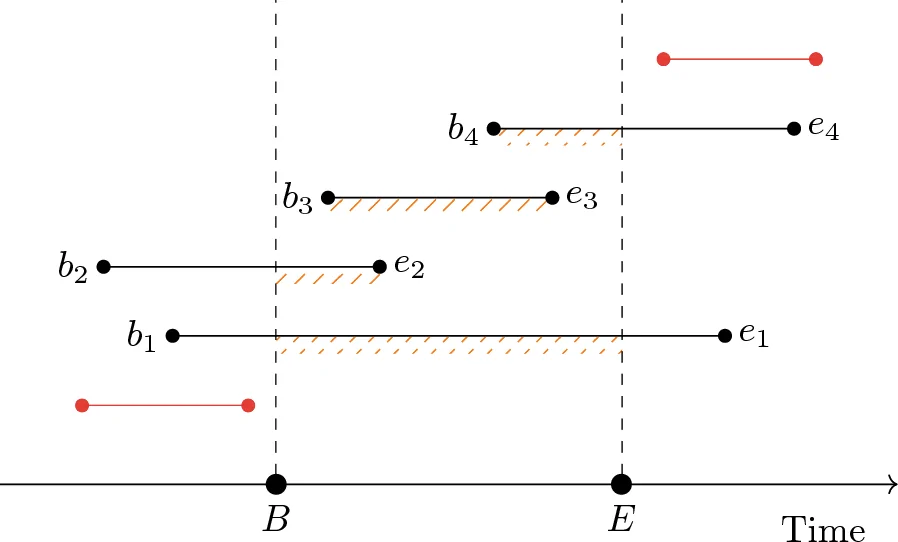
Illustration of aggregation scenarios for continuous drug administration intervals $(b_a, e_a)$ for $a\in \{1,2,3,4 \}$, within an aggregation window (*B*, *E*). Non-intersecting intervals (red) do not contribute to the predictor variables, whereas yellow-shaded intersections indicate the time periods contributing information

The continuous intravenous administration of the pharmaceutical agent requires explicit processing of the dosing period. A single continuous drug administration *a* has a start time $b_a$ and end time $e_a$, resulting in the single dosing interval $(b_a, e_a)$. For the calculation of the cumulative administered doses, it is important to know which aggregation time interval (*B*, *E*) with start and end time *B* and *E* is relevant in a specific case. We have two differing cases. In case 1 for the aggregation of a non-vancomycin drug treatment prior to time point $t_{\text {dose}}$ (e.g. norepinephrine administration), a 24-hour time window was chosen to define aggregation window $(B, E) = (t_{\text {dose}} - 24h, t_{\text {dose}})$. In case 2, the aggregation of vancomycin treatment between time point $t_{\text {dose}}$ and time point $t_{\text {meas}}$ was conducted, however resulting in a different interval length $(B, E) = (t_{\text {dose}}, t_{\text {meas}})$. The calculation of cumulative doses, however, works analogously. This is necessary, because continuous infusion may begin before, or extend beyond, the boundaries of the relevant aggregation window (B, E), so that only the portion overlapping the window should contribute. To enable more accurate integration of drug administration as predictors, a constant infusion was assumed. Figure 2 illustrates the scenarios requiring consideration. In general, for any administration with a non-empty overlap between the dosing interval $(b_a, e_a)$ and the aggregation window (*B*, *E*), specifically when $e_a > B$ and $b_a < E$, the model-relevant interval is defined as the intersection $(b_a, e_a)\cap (B, E) = (\max (b_a, B), \min (e_a, E))$. Its duration, $\min (e_a, E) - \max (b_a, B)$, is depicted as the shaded segments in Fig. 2. The cumulative dose was finally obtained by multiplying each overlapping interval’s duration by the corresponding administration rate and summing across all relevant intervals.

### Data pre-processing

In order to enhance prediction performance and generalizability, the aggregated and pre-selected data are pre-processed, before being used in the machine learning algorithms. Predictors with zero variance and more than *30%* missing values are being removed. Creatinine clearance was calculated using the Cockcroft-Gault formula. For patients with missing serum creatinine, a plausible population value of 0.9 mg/dL was imputed beforehand, consistent with the approach used in the benchmarking PopPK models, ensuring that both approaches are exposed to the same information constraints and thereby allowing a fair comparison of predictive performance. These steps must be regarded as integral elements of the modelling pipeline. The remaining missing values are imputed by means of median imputation. These steps must be regarded as integral elements of the modelling pipeline. To prevent overly optimistically or pessimistically biased estimates of generalization error, all pre-processing was therefore embedded within the resampling procedure, namely the repeated nested cross-validation.

### Modelling and evaluation methods

The aim of this work was to model the concentration $\textbf{y} = f(\textbf{X})$ using a function *f* that characterizes the relationship between the outcome vector of concentrations $\textbf{y} \in \mathbb {R}^{n}$ and the set of predictors $\textbf{X} \in \mathbb {R}^{n \times p}$ of all patients, and to enable prediction of a new observation $\widehat{y}_i = f(\widetilde{\textbf{x}}_i)$ based on the clinical information $\widetilde{\textbf{x}}_i$ of an unseen patient. Statistical and machine learning approaches exhibit complementary strengths, such as robustness and non-linear modelling capacity, as well as limitations including noise sensitivity and limited interpretability. Accordingly, no single method is universally optimal. In this study, the relatively small sample size *n* compared with the number of potentially influential predictors *p* motivated the selection of robust, regularized machine learning models. Specifically, elastic net regression, random forests, and extreme gradient boosting (XGBoost) were employed, as these methods provide stable and reliable predictions in small-sample, high-dimensional settings despite differing underlying principles.

The contemporary approach for predicting concentrations is based on pharmacokinetic modelling of the time-concentration curve. Due to the sparse routinely captured data, there was no feasible foundation for our own PK model estimations. Concentration-time profiles simulated from existing PopPK models were used as a baseline to benchmark our modelling approach. All analyses were conducted using the programming language R [42] and program code will be openly available in a github-repository^2^ and also discloses all software packages used in the modelling. In order to comprehend the general modelling workflow, one may refer to the flowchart in the supplementary material. The proposed methods are presented briefly. Additional methodological details are provided in the supplementary material, together with references to advanced theoretical literature.

#### Elastic net

Linear models of the form $\textbf{y} = f(\textbf{X}) = \beta _0 + \sum _{j=1}^{p} x_j \beta _j$ attempt to model the relationship between the outcome and the predictors with a linear combination using regression coefficients $\boldsymbol{\beta } = (\beta _1,\dots ,\beta _p)'$, but estimation becomes challenging when the sample size *n* is small relative to the number of potentially influential predictors *p*. As *p* increases, higher correlation among predictors becomes more likely, resulting in numerically unstable parameter estimates. In many applications, it is desirable to identify a parsimonious subset of predictors that preserves predictive performance while enhancing interpretability and practical applicability. To address these issues, elastic net regularization [43] extends linear regression by introducing penalties on the regression coefficients $\boldsymbol{\beta }$. Specifically, the elastic net combines ridge and lasso regression through a convex combination of $L_2$ and $L_1$ penalties, controlled by an overall regularization parameter *λ > 0* and a mixing parameter $\alpha \in (0,1]$ resulting in the estimated coefficients

$$\begin{aligned} \widehat{\mathbf {\beta }} = \arg \min _{ \beta _0, \beta } \Bigg \{&\frac{1}{n} \sum _{i=1}^{n} \left( y_i - \textbf{x}_i' \mathbf {\beta } \right) ^2 + \\ &\lambda \sum _{j=1}^{p} \left( \alpha |\beta _j|_1 + \frac{1-\alpha }{2} |\beta _j|_2^2 \right) \Bigg \}. \end{aligned}$$

The parameters *λ* and *α* have to be tuned using resampling to optimize predictive performance while maintaining model sparsity. A prediction $\widehat{y}$ for a newly observed individual with predictor set $\widetilde{\textbf{x}}$ are easily calculated by $\widehat{y} = \widehat{\beta }_0 + \sum _{k=1}^{p} x_j \widehat{\beta }_k$.

#### Random forest

A random forest regression model is an ensemble method that builds upon regression trees to model complex, non-linear relationships between predictors and a continuous outcome. A single regression tree partitions the predictor space into disjoint regions using recursive binary splits and predicts the outcome within each terminal node as the mean response $\widehat{f}_{\text {tree}}(\widetilde{\textbf{x}})$ of the observations in that region, obtained by minimizing the residual sum of squares (RSS). random forests, originally introduced by [44], extend this concept by constructing an ensemble of *B* regression trees $\{f_b\}_{b=1}^B$ and aggregating their predictions. Each tree is trained on a bootstrap sample drawn with replacement from the original dataset $D = \{(\textbf{x}_i, y_i)\}_{i=1}^n$, following the bootstrap aggregating (bagging) principle [45]. In addition, at each split only a randomly selected subset of predictors is considered, which reduces correlation between individual trees. For a new observation $\widetilde{\textbf{x}}$ the random forest prediction is obtained by averaging the predictions of all individual trees,

$$\begin{aligned} \widehat{y} = \frac{1}{B}\sum _{b=1}^{B} \widehat{f}^{(b)}_{\text {tree}}(\widetilde{\textbf{x}}). \end{aligned}$$

By aggregating many weakly correlated trees, random forest regression substantially reduces variance and overfitting compared with a single regression tree, while maintaining low bias. To encourage model parsimony, extensions of tree-based methods have introduced regularization of the splitting criterion, for example by penalizing the RSS with a tuning parameter that discourages unnecessary splits [46]. In random forests, several hyperparameters affect model complexity and performance and are typically set to established defaults or optimized using resampling-based tuning procedures.

#### XGBoost

Gradient boosting methods construct tree-based ensembles sequentially, with each new tree fitted to the residual errors of the current model [[45], p. 337 ff.]. This iterative procedure enables flexible modelling of complex, non-linear relationships. eXtreme Gradient Boosting (XGBoost), introduced by [47], extends classical gradient boosting by incorporating explicit regularization and improving computational efficiency. Formally, XGBoost iteratively predicts $\widehat{y} = \phi (\textbf{x})= \sum _{t=1}^{M} \eta f_t(\textbf{x})$ with trees $f_t$ in iteration *t*, by minimizing a regularized squared error loss

$$\begin{aligned} \mathcal {L}(\phi ) =&\sum _{i=1}^{n} \left( y_i - \bigl (\widehat{y}_i^{(t-1)} + f_t(\textbf{x}_i)\bigr )\right) ^2 + \\&\gamma T + \frac{1}{2}\lambda \sum _{j=1}^{T} w_j^2. \end{aligned}$$

To prevent overfitting, the parameters *γ* and *λ* control the number of leaves *T* in the tree and their corresponding leaf weights $w_j$ penalizing model complexity. Predictions are updated sequentially via

$$\begin{aligned} \widehat{y}_i^{(t)} = \widehat{y}_i^{(t-1)} + \eta f_t(\textbf{x}_i), \end{aligned}$$

where $\eta \in (0,1]$ is the learning rate controlling the contribution of each tree. The final prediction after *M* iterations is given by $\widehat{y}_i = \widehat{y}_i^{(0)} + \sum _{t=1}^{M} \eta f_t(\widetilde{\textbf{x}}_i)$. The hyperparameters of the XGBoost model, including the number of boosting iterations *M*, learning rate *η*, maximum tree depth, subsampling rate, and regularization parameters *γ* and *λ*, need to be optimized using resampling-based tuning approaches to balance predictive accuracy and generalization.

#### Hyperparameter

The predictive qualities of a ML-algorithm depend not only on the quality of the data set, the choice of a model, but also heavily on the set of hyperparameter. Tuning of these parameters in ML-algorithms are an integral component of the modelling procedure itself and is usually computationally expensive. To limit complexity, only hyperparameters with meaningful effects and well-established default ranges were tuned, following the literature review of [48] for elastic net, random forest, and XGBoost. Additional parameters were included to account for the specific characteristics of our data, such as small sample size, high dimensionality, and correlated predictors, particularly those governing regularization.

#### Performance Evaluation via Nested Resampling

A simple train-test split for model evaluation provides only a single, biased estimate and does not adequately reflect variability of the generalization error, particularly in smaller datasets. To obtain a more reliable estimate, a stratified 5-times repeated 15-fold cross-validation procedure as described by [49], was used. In this approach, an inner cross-validation is conducted within each outer training partition to identify optimal hyperparameters. The tuned models are subsequently evaluated on the corresponding outer test set. Stratification was conducted using three clinically motivated concentration strata, subtherapeutic (*<15* mg/L), therapeutic (*15-25* mg/L), and supratherapeutic (*>25* mg/L), to ensure a valid estimation of the model’s generalization error. This design guarantees strict separation between training and testing data for every modelling step, thereby preventing data leakage and reducing the risk of optimistic bias in performance estimation enables a rigorous assessment of model generalizability.

#### Performance Metrics

To comprehensively compare model performance across resampled data subsets, multiple complementary performance metrics were employed, as no single statistic sufficiently captures all relevant aspects of predictive performance. These metrics were based on the difference between observed and predicted concentrations. The root mean squared error $\text {RMSE} = \sqrt{\frac{1}{n} \sum _{i=1}^{n} (y_i - \widehat{y}_i)^2}$ was used to penalize larger deviations more strongly. The mean absolute error $\text {MAE} = \frac{1}{n} \sum _{i=1}^{n} |y_i - \widehat{y}_i|$ was reported as a more interpretable measure of the average absolute deviation. To quantify the explained variance of the predictions, the coefficient of determination was computed as $R^2 = 1 - (\sum _{i=1}^{n} (y_i - \widehat{y}_i)^2)/\sum _{i=1}^{n} (y_i - \bar{y})^2)$. Together, these metrics provide a comprehensive and interpretable assessment of model accuracy and generalizability across all resampled datasets.

#### Simulation from PopPK-Models

To benchmark our ML-based predictions against established pharmacokinetic knowledge, we considered seven previously published vancomycin PopPK models implemented in TDMx. These models are widely used to support TDM-based dosing recommendations and therefore provide a relevant reference for evaluating alternative predictive approaches. Although originally developed in distinct patient cohorts, they currently represent the most established and practically applied framework for guiding vancomycin dosing in clinical practice. Within this set of reference models, several studies adopted a two-compartment description of vancomycin pharmacokinetics, including the models by [50] (hospitalized patients, 1812 patients and 2765 samples) and [51] (hospitalized patients, 398 patients and 1557 samples), [52] (critically ill patients with post-sternotomy mediastinitis, 30 patients and 359 samples), and [53] (hematopoietic stem-cell transplantation patients, 95 patients and 285 samples). In contrast, [54] (ICU patients, 191 patients and 569 samples), [55] (critically ill patients, 206 patients and 579 samples), and [56] (obese patients, 29 patients and 93 samples) identified a one-compartment structure as the most appropriate representation for their respective cohorts.

## Results

### Cohort characteristics

Table 1 reflects the utilized clinical predictors and provides coarse information about the distribution and missing values. The cohort included middle-aged patients (mean age 59.8 years), predominantly male (*62%*), with a moderately elevated BMI (mean 30.5 kg/m$^{2}$), indicating a population at risk for altered vancomycin pharmacokinetics due to changes in volume of distribution and renal clearance. Renal function showed pronounced heterogeneity, with creatinine clearance ranging from 12.5 to 258.6 L/h, underscoring the need for individualized dosing. ICU-specific factors, such as vasopressor use, fluid administration, and ventilation further reflect substantial variability in hemodynamic and organ function. Notably, relevant anthropometric variables, such as height weight or BMI, exhibited considerable missingness, limiting their utility for pharmacokinetic modelling and shifting emphasis toward more consistently available variables. Mean vancomycin concentrations after initial dosing were elevated at approximately 24 mg/L.

**Table 1:** Descriptive table of pre-selected clinical features aggregated according to domain expertise

**Feature**	**Mean/%**	**Standard deviation**	**Range**	**No. missing**
**Baseline characteristic**				
Female (gender) (yes/no: 1/0)	38 %		{0,1}	0
Age (in years)	59.84	14.64	[21–87]	0
Height (cm)	174.12	7.79	[160–188]	15
Weight (kg)	92.7	25.37	[50–175]	14
BMI (kg/$\text {m}^2$)	30.54	8.16	[18.8–62.5]	15
**Comorbidities**				
Chronic kidney disease (yes/no: 1/0)	12 %		{0,1}	0
Diabetes (yes/no: 1/0)	36 %		{0,1}	0
**Vital parameters**				
SOFA Score	10.5	2.03	[6–16]	16
Mean invasive blood pressure (mmHg)	81.1	12.7	[28–118.5]	6
Horowitz quotient (mmHg)	297.12	139.64	[129.09–817.38]	6
Mechanical ventilation (yes/no: 1/0)	44 %		{0,1}	0
Dialysis (yes/no: 1/0)	27 %		{0,1}	0
**Laboratory parameters**				
Serum creatinine (mg/dL)	1.47	0.89	[0.25–4.66]	3
Creatinine clearance (L/h)	87.08	56.4	[12.52–258.57]	0
Blood urea nitrogen (mg/dL)	30.51	16.33	[8–79.9]	3
Total bilirubin (mg/dL)	1.06	1.15	[0.19–6.03]	3
Glutamic oxaloacetic transaminase (U/L)	177.52	483.85	[12.5–2951]	3
Prothrombin time (Quick) (%)	67.97	18.25	[22–100]	5
Hemoglobin (g/dL)	9.84	1.89	[7.49–15]	1
Platelet count ($10^3$/*μ*L)	228.5	155.59	[28.83–884]	4
Nucleated red blood cells ($10^3$/*μ*L)	0.38	1.09	[0–7.25]	4
Red cell distribution width (%)	16.9	3.29	[12.3–29.34]	4
Total protein (g/dL)	5.31	1.09	[2.2–7.5]	7
C-reactive Protein (mg/dL)	16.68	10.99	[1.11–44]	3
Procalcitonin (ng/mL)	10.29	19.23	[0.07–93.73]	7
**Treatment course**				
Time since admission (h)	176.72	163.45	[19.1–640.73]	0
Time since first dose (h)	31.99	14.97	[13.37–88.83]	0
Administered vancomycin (cumulative) (g)	1.9	0.76	[0–3.83]	0
Administered norepinephrine (cumulative) (mg)	26.99	40.45	[0–210.22]	0
Total fluid intake (mL)	1600.57	1221.03	[0–5701]	0
Urine output (24h) (mL)	487.97	703.02	[0–2830]	0
Concentration (mg/L)	24.07	10.65	[2.29–54.9]	0

The table reports empirical distributions using measures of central tendency (arithmetic mean for continuous variables; percentages for binary variables),

measures of dispersion (standard deviation, minimum, and maximum), and the number of missing values. For binary variables, values represent

proportions (e.g., Female (gender) (yes/no: 1/0) = 38% indicates that 38% of patients are female), **bold** formatting is used to indicate section headings within the tables and has no statistical significance

### Performance results

**Fig. 3:**
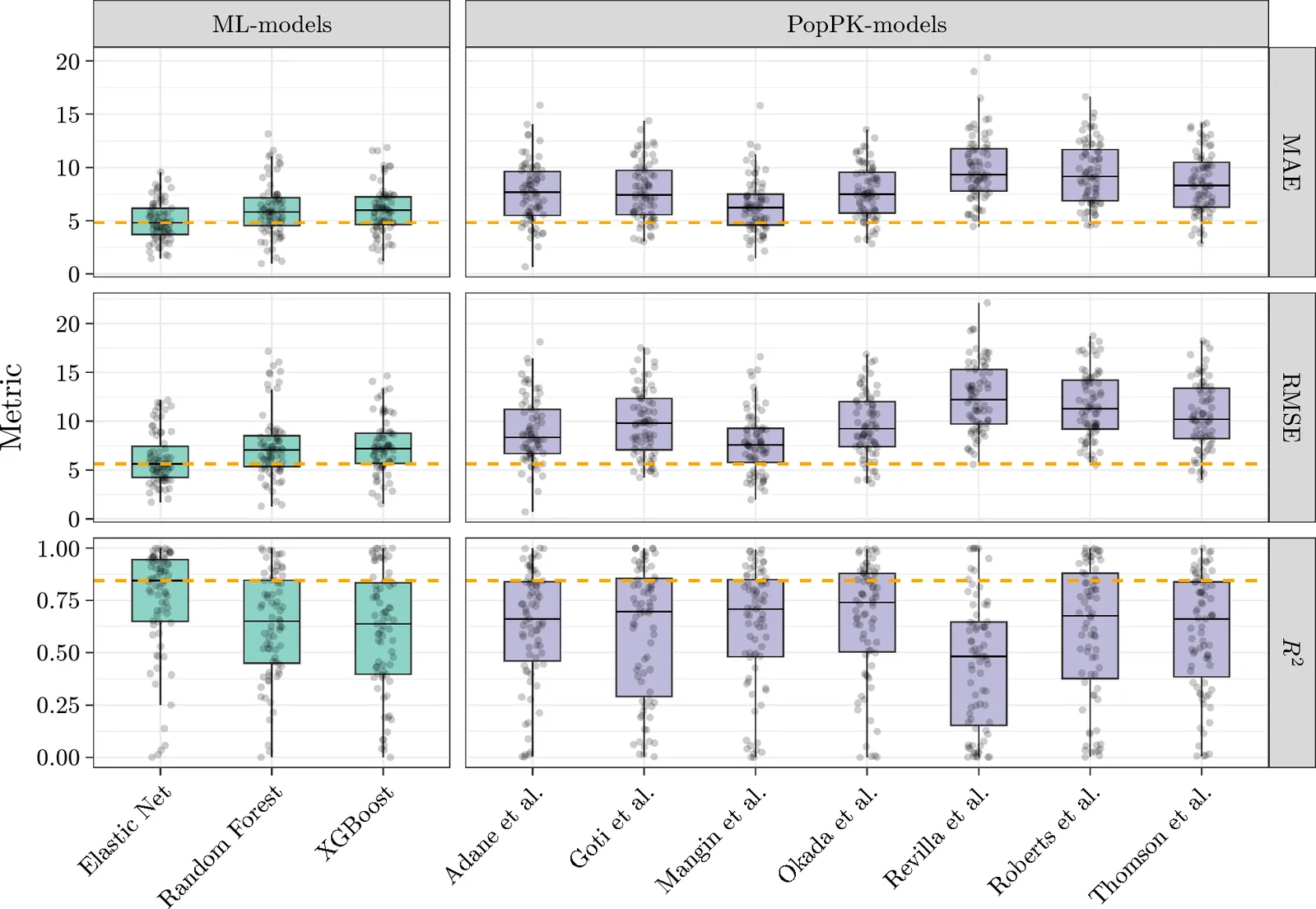
Distribution of nested cross-validation performance metrics (MAE, RMSE, and $R^2$) on the outer resampling level, shown as boxplots (median, 25th and 75th percentiles, with whiskers at 1.5-times the interquartile range) with individual values for competing PK models and the proposed models. The dashed orange line indicates the median of the best-performing model

Figure 3 illustrates the performance differences between the three ML and seven PopPK models. Both approaches generally captured vancomycin concentrations, highlighting the challenges posed by the heterogeneity of vancomycin dynamics in septic patients. PopPK models, however, showed greater variability and occasional high-error outliers in some resampled subsets, reflecting the constraints of fixed structural assumptions and potentially relevant missing information modelling complex patient variability.

Across all three metrics ML models generally exhibit less dispersed distributions of metrics with lower median errors and higher explained variance, indicating more consistent and accurate predictions. The elastic net model in particular shows the narrowest interquartile range and the highest median $R^2$ among ML methods, highlighting its robustness.

These results may indicate the advantage of ML approaches in providing more accurate and stable individualized vancomycin concentration predictions in septic patients. For instance, taking a look at results in Table 2, the best-performing PopPK model (Mangin et al.) showed a MAE of 6.38 mg/L, a RMSE of 7.83 mg/L, and an $R^2$ of 0.62. In comparison, the elastic net improved predictive performance, reducing the MAE to 4.98 mg/L, the RMSE to 6.19 mg/L, and increasing $R^2$ to 0.75, highlighting the advantage of ML approaches in integrating diverse patient-specific and dynamic clinical variables to enable more precise individualized dosing. The superior performance metric $R^2$ of the elastic net relative to the other ML models may reflect the presence of an underlying linear relationship and the model’s suitability for small-sample settings.

**Table 2:** Descriptive statistics (mean and standard deviation) of model performance metrics across all models

	**Mean (Standard deviation)**		
	**MAE**	**RMSE**	$R^2$
**PopPK-models**			
Adane et al.	7.62 (2.86)	8.97 (3.33)	0.63 (0.28)
Goti et al.	7.75 (2.71)	9.8 (3.37)	0.6 (0.32)
Mangin et al.	6.38 (2.56)	7.83 (3.14)	0.62 (0.29)
Okada et al.	7.65 (2.49)	9.54 (3.25)	0.66 (0.28)
Revilla et al.	9.91 (3.08)	12.46 (3.53)	0.44 (0.31)
Roberts et al.	9.4 (2.9)	11.6 (3.39)	0.6 (0.33)
Thomson et al.	8.57 (2.82)	10.57 (3.47)	0.6 (0.28)
**ML-models**			
Elastic net	*4.98 (1.81)*	*6.19 (2.64)*	*0.75 (0.26)*
Random forest	6.03 (2.51)	7.48 (3.47)	0.63 (0.25)
XGBoost	6.04 (2.25)	7.46 (2.81)	0.6 (0.29)

Mean metric values and their standard deviations for the best-performing models are shown in *italic*, **bold** formatting is used to indicate section headings within the tables and has no statistical significance

In order to approximate the potential impact of this alternative prediction strategy, the accuracy of the strategy can be evaluated on a coarser concentration scale. When the goal is simply to determine whether a patient will exhibit a low, target, or high concentration, both observed and predicted values can be categorized using predefined thresholds. Following the recommendation of [57], the target range of *15-25* mg/L is used here for illustration.

**Fig. 4:**
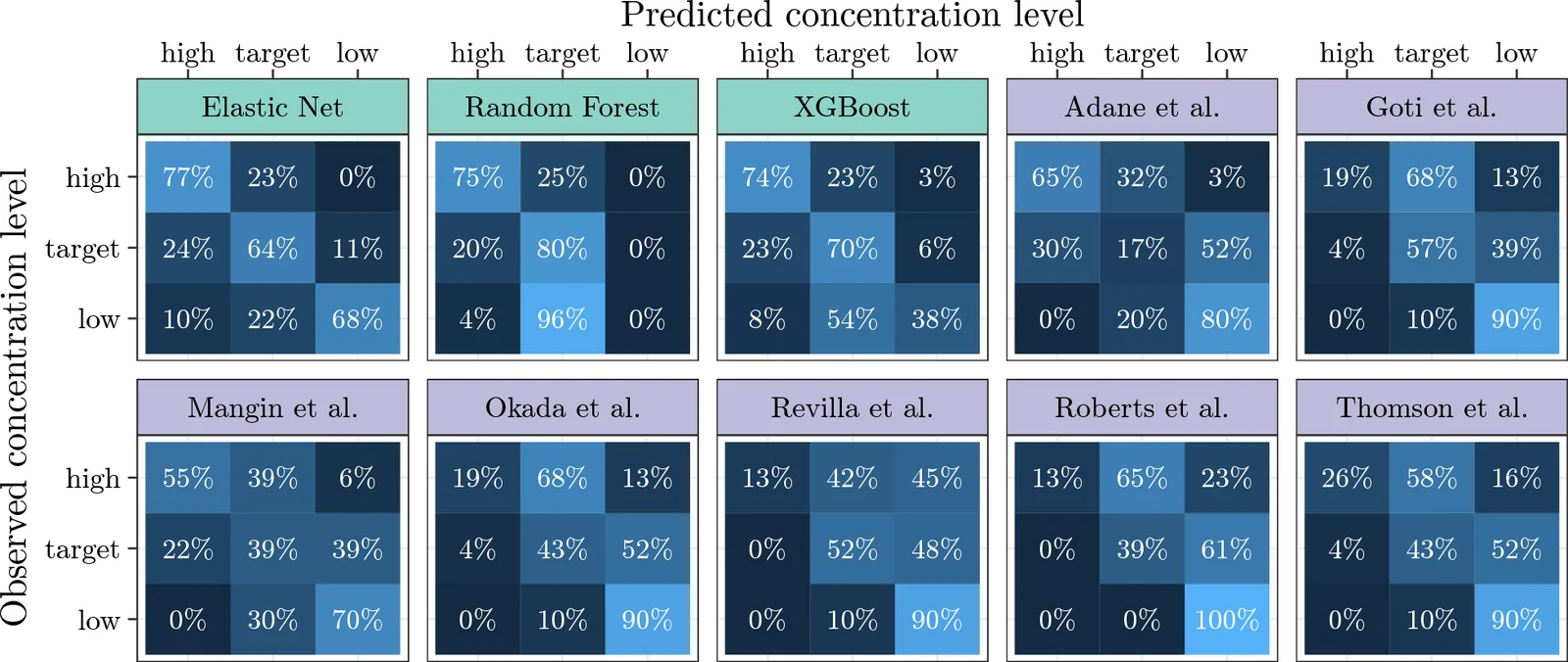
Modified confusion matrix for 1408 resampled observations (sub-therapeutic: 220; therapeutic: 506; supra-therapeutic: 682) across outer cross-validation folds, showing row-wise classification accuracy for three concentration levels. Because the population PK models are deterministic and do not simulate random effects, predictions for a given patient are identical across all resamples

Figure 4 presents a modified confusion matrix for the resampled observations and their predictions across all outer cross-validation folds, categorized into the three levels: subtherapeutic, therapeutic, and supratherapeutic. Each row shows the percentage of correctly classified cases within that category, representing the proportion of patients accurately predicted. The PopPK models demonstrate good accuracy at low concentrations, correctly classifying *70-100%* of patients as sub-therapeutic. However, their performance deteriorates at high concentration levels, where only *13-65%* of cases are correctly categorized as supra-therapeutic. In our septic cohort, high initial concentrations are common and are reliably captured only by the ML methods. Random forest and XGBoost correctly detect high (*75%* and *74%*) and target (*80%* and *70%*) concentrations but perform poorly for low concentrations, whereas the elastic net achieves adequate accuracy across all three concentration levels, with consistently over *64%*. In particular, the random forest fails to reliably identify sub-therapeutic concentrations, likely due to the limited number of patients with low concentrations and the lack of the strong regularization required in small-sample settings. The model by Mangin et al. also demonstrates acceptable performance, correctly identifying at least *39%* of categories. Regarding misclassification patterns, the elastic net primarily makes errors between adjacent concentration levels, with rates ranging from *11%* (classifying supra-therapeutic concentrations as therapeutic) to *24%* (classifying therapeutic concentrations as supra-therapeutic). Despite its overall strong performance, approximately *10%* of resampled sub-therapeutic test observations are misclassified as supra-therapeutic. These patterns may be relevant when considering the clinical application of the model.

### Final model

To obtain a deployable and interpretable prediction model, we applied the same tuning and fitting procedure used in the inner resampling to the full dataset. Based on its superior generalization performance compared with the PopPK models, the elastic net was selected and fitted as the final model. The final tuning procedure yielded a hyperparameter configuration of penalization term *λ = 0.89* and mixture parameter *α =0.3*, reflecting a balance between ridge and lasso penalization. Using this configuration, the elastic net model was then fitted to the full dataset. Table 3 presents the regression coefficients of predictors retained by the elastic net, demonstrating clinically coherent feature selection. Coefficients are reported on both unstandardized and standardized scales. Coefficients for unstandardized predictors enable clinical interpretation and practical prediction by showing the change in outcome per unit change in each predictor, while coefficients for standardized predictors reveal the relative importance of predictors within the model.

**Table 3:** Final regression coefficients $\widehat{\beta }_k > 0$ of the elastic net, for unstandardized (unstd.) and standardized (std.) predictor scales

	$$\widehat{\beta }_k$$	
	**unstd.**	**std.**
(Intercept)	$\phantom {-}5.606$	$\phantom {-}24.065$
**Baseline characteristic**		
Female (gender)	$\phantom {-}2.255$	$\phantom {0}\phantom {-}1.098$
Age	$\phantom {-}0.024$	$\phantom {0}\phantom {-}0.344$
Weight	$\phantom {-}0.006$	$\phantom {0}\phantom {-}0.129$
BMI	$\phantom {-}0.013$	$\phantom {0}\phantom {-}0.094$
**Comorbidities**		
Chronic kidney disease	$\phantom {-}8.753$	$\phantom {0}\phantom {-}2.912$
**Vital parameters**		
SOFA Score	*-0.410*	$\phantom {0}-0.722$
Horowitz quotient	*-0.002*	$\phantom {0}-0.231$
Mechanical ventilation	*-2.302*	$\phantom {0}-1.148$
**Laboratory parameters**		
Serum creatinine	$\phantom {-}3.028$	$\phantom {0}\phantom {-}2.628$
Creatinine clearance	*-0.032*	$\phantom {0}-1.821$
Glutamic oxaloacetic transaminase	$\phantom {-}0.005$	$\phantom {0}\phantom {-}2.218$
Nucleated red blood cells	$\phantom {-}0.903$	$\phantom {0}\phantom {-}0.955$
Red cell distribution width	$\phantom {-}0.181$	$\phantom {0}\phantom {-}0.573$
Procalcitonin	*-0.108*	$\phantom {0}-1.958$
**Treatment course**		
Time since first dose	$\phantom {-}0.133$	$\phantom {0}\phantom {-}1.982$
Administered vancomycin (cumulative)	$\phantom {-}5.717$	$\phantom {0}\phantom {-}4.353$
Administered norepinephrine (cumulative)	$\phantom {-}0.041$	$\phantom {0}\phantom {-}1.641$
Urine output (24h)	*-0.002*	$\phantom {0}-1.526$

**Bold**formatting is used to indicate section headings within the tables and has no statistical significance

Coefficients of standardized predictors reveal three conceptual groups of predictive importance. Drug exposure and renal function dominate the model. The cumulative vancomycin dose ($\widehat{\beta } = 4.353$) emerges as the strongest predictor, while information about renal function like chronic kidney disease ($\widehat{\beta } = 2.912$), serum creatinine ($\widehat{\beta } = 2.628$) or creatinine clearance ($\widehat{\beta } = -1.821$) collectively demonstrate the critical role of kidney function in vancomycin pharmacokinetics. This pattern aligns with vancomycin’s predominant renal elimination also reflected in PopPK models and a narrow therapeutic range. A second group comprises temporal and hemodynamic factors: time since first dose ($\widehat{\beta } = 1.982$), cumulative norepinephrine ($\widehat{\beta } = 1.641$) and urine output ($\widehat{\beta } = -1.526$) reflect the drug accumulation and the impact of cardiovascular instability on drug clearance. Notably, hepatic dysfunction (indicated by GOT, $\widehat{\beta } = 2.218$) and inflammatory response (measured by PCT, $\widehat{\beta } = -1.958$) contribute substantially, suggesting complex organ interactions in septic patients. Critical illness severity markers form a third group with consistent negative associations: SOFA score ($\widehat{\beta } = -0.722$), mechanical ventilation ($\widehat{\beta } = -1.148$) and Horowitz quotient ($\widehat{\beta } = -0.231$) potentially reflect increased volume of distribution or augmented renal clearance. Baseline patient characteristics (age, weight, BMI, gender) and hematological markers demonstrate minimal relative importance, indicating that acute pathophysiological changes outweigh static demographic factors in this critically ill population.

**Fig. 5:**
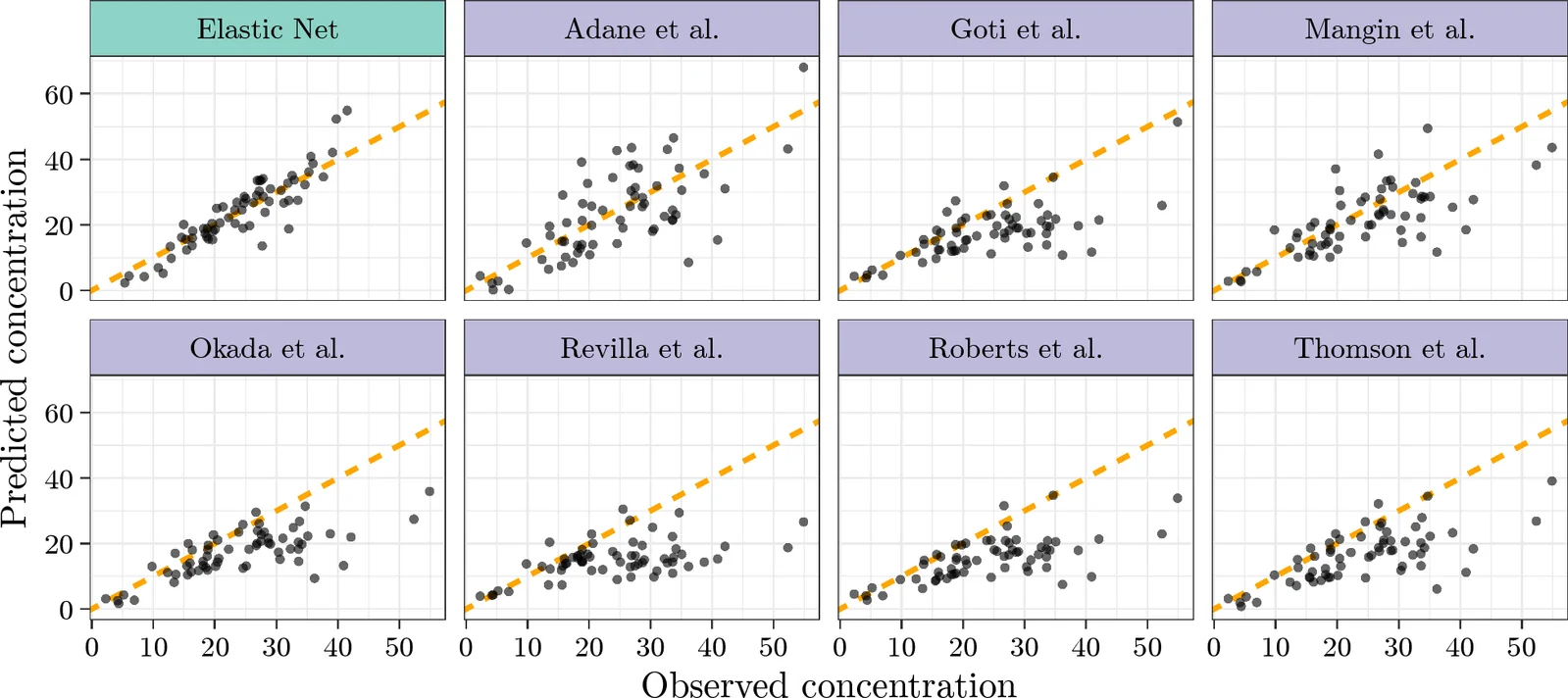
Scatterplots of final predicted concentration against observed concentration of the winning ML-model of the elastic net and the 7 competing PopPK-models for the entire observed dataset

Based on these coefficients the fitted concentrations, a prediction of all observed concentratons is illustrated in Fig. 5. The figure illustrates the goodness of fit by presenting a pair of the aforementioned values of one patient in each point.

It can be seen, that the elastic net shows a desirable narrow point cloud on the bisecting line, which shows only a small difference between observed and predicted concentration. It can be noticed, that the fitted quality show few extreme values, especially with very high observed concentration. This could be caused by unexplained variance due to missing predictor information. This seems still less significant, than when we would have used only PopPK models for the prediction. The PopPK models exhibit more variability in predictions and a less confident accuracy overall. While exhibiting this dispersion, models for the populations of [52] and [56] show little systematic bias for specific concentration levels and seem to work decently well for our sepsis cohort although patient characteristics were still different. This demonstrates the robust quality and practical utility of these models in providing usable dosing support.

## Discussion

This study demonstrates that ML-based prediction of vancomycin serum concentrations offers clear advantages over conventional PopPK models in patients with sepsis admitted to an ICU. A comparison of the PopPK models implemented in TDMx revealed distinct performance patterns across the concentration range. While TDMx models predicted low concentrations with reasonable accuracy, their precision declined at higher concentrations. In contrast, the ML models demonstrated superior performance in the supratherapeutic range. Among the evaluated approaches, the elastic net regression model demonstrated the most balanced performance across subtherapeutic, therapeutic, and supratherapeutic concentrations, thus making it the most suitable candidate for integration into clinical decision support. Notably, our ML approach, utilizing exclusively routinely available clinical data, enabled the accurate and interpretable prediction of concentrations prior to the availability of TDM results. These findings underscore the potential of ML to enhance the precision of early dosing and to support individualized antimicrobial therapy in sepsis.

Beyond the predictive performance, the predictors retained by the elastic net reflect clinically plausible determinants of vancomycin exposure and closely mirror known pharmacokinetic principles. The observed predominance of cumulative dose and renal function is consistent with the predominantly renal elimination of vancomycin and its narrow therapeutic range, as predicted by established PopPK models [55, 58]. However, the model also retained several variables that are not typically incorporated into pharmacometric models. This suggests that ML captures broader physiological dimensions relevant to drug disposition in sepsis.

Temporal and hemodynamic variables, including the time since the initial dose was administered, the requirement of norepinephrine, and urine output, are likely indicative of drug accumulation and the impact of cardiovascular instability on renal clearance. Vasopressor dependency, in particular, has been identified as a clinically significant marker of impaired organ perfusion and altered regional blood flow [59]. This, in turn, may indirectly reduce vancomycin clearance. Concurrently, the presence of certain markers, including NRBCs [60], RDW [61, 62], liver transaminases (GOT) [63, 64], and PCT [65], have been observed to correlate with global physiological stress, systemic inflammation, hypoxia, and evolving organ dysfunction. These variables are not directly implicated in the metabolism of vancomycin; however, they likely encapsulate integrative aspects of disease severity, inflammatory burden, and circulatory instability that are challenging to formalize within the framework of traditional PopPK models. The negative associations observed for SOFA score, mechanical ventilation, and the Horowitz quotient are more likely explained by fluid resuscitation intensity and an expanded volume of distribution in more severely ill patients [66, 67] than by a direct effect on renal clearance, particularly given that renal function is already captured separately through creatinine and creatinine clearance. The effect size for the Horowitz quotient is notably small (with an estimated coefficient of 0.002), with a 100-unit change corresponding to only a 0.2 mg/L shift in predicted concentration, and should not be over-interpreted. More generally, SOFA score, mechanical ventilation, vasopressor use, and the Horowitz quotient likely reflect complex, overlapping pathophysiological processes that a regularized model fitted on 64 patients cannot fully disentangle. Individual coefficients, particularly smaller ones, should therefore be interpreted with caution. Although dialysis status was considered as a candidate predictor, it was not retained in the final model, likely because it overlaps considerably with other renal markers already included (serum creatinine, creatinine clearance, and chronic kidney disease) and because only a minority of patients in our cohort were on dialysis. As dialysis can influence vancomycin clearance independently of creatinine-based estimates of renal function, we regard it as a variable worth revisiting in a larger cohort with more dialysis patients. Conversely, baseline demographic characteristics exhibited minimal significance, suggesting that acute pathophysiological alterations supersede static patient characteristics in this critically ill population [10].

A key advantage of the model lies in the clinical interpretability of the unstandardized coefficients. These effect sizes illustrate how individual clinical factors translate into quantitative changes in predicted concentration. For example, chronic kidney disease increased predicted concentration by 8.753 mg/L, and each gram of vancomycin administered added 5.717 mg/L, allowing concentration differences exceeding 15 mg/L between patients to be explained by these factors alone. Renal function parameters further enabled quantitative adjustment: each 1 mg/L increase in serum creatinine increased concentration by 3.028 mg/L, while each 1 mL/min decrease in creatinine clearance added approximately 0.032 mg/L. Modifiers of critical illness were similarly impactful, with mechanical ventilation reducing predicted concentration by 2.302 mg/L and each SOFA point decreasing it by 0.410 mg/L. In severely ill patients (e.g., SOFA *≥ 10*), these effects become clinically substantial. The coefficient for body weight was positive but negligible in size (0.006 mg/L per kilogram), corresponding to a shift of only about 0.12 mg/L across a 20 kg difference in weight, a difference too small to be clinically meaningful. We do not interpret this as a genuine physiological effect, since a positive weight-concentration association runs counter to expected pharmacokinetic behavior, given that higher weight is typically linked to a larger volume of distribution and lower concentrations. This coefficient more likely reflects residual correlation with other retained predictors, particularly administered vancomycin dose, which is often prescribed on a weight-adjusted basis, rather than an independent effect of weight itself. In principle, these coefficients could allow clinicians to estimate expected concentration changes from specific clinical conditions or interventions and thereby support individualized dosing decisions.

The comparison with established PopPK models further illustrates that, while these models provide reasonable predictions without marked systematic bias, they exhibit greater variability across concentration ranges. The application of the elastic net approach led to a reduction in this dispersion and an enhancement in the degree of agreement between the observed and predicted concentrations. This outcome is presumably attributable to the capacity of ML models to integrate multidimensional clinical information and to capture complex, non-linear interactions that are characteristic of sepsis-related alterations in drug handling. Together, these findings demonstrate how ML-based approaches can complement pharmacometric modelling by incorporating dynamic physiological information that is not easily parameterized in traditional models, thereby improving early and individualized dosing support in critically ill septic patients.

Despite promising findings, several limitations apply. This was a single-center study with a comparatively small cohort of 64 patients, which limits generalizability and carries a risk of overfitting. We mitigated this risk through repeated nested cross-validation with all pre-processing embedded in the resampling loop, and by restricting the analysis to lower-variance, complexity-controlled learners; notably, the best-performing model was also the most strongly regularized (elastic net), and performance was consistent across the repeated outer folds. Nonetheless, nested cross-validation reflects internal validity only and cannot substitute for external validation on an independent cohort, which was not performed here because no suitable external dataset was available. The present results should therefore be viewed as a single-center proof of concept, and external validation in larger, ideally multi-center cohorts is an essential next step to test whether the predictors and effect sizes identified here generalize more broadly. An additional limitation concerns the aggregation of time-varying predictors, which relied solely on the mean, primarily to limit the number of predictors relative to our small sample size. This comes at a cost, as a mean can obscure a patient’s underlying trajectory: a patient with recently improving renal function, for example, may have largely restored vancomycin clearance despite a mean creatinine similar to, or higher than, that of a patient with stable, moderately impaired renal function. As a simple average, the mean neither reflects the direction of change within the window, which a temporal slope or a first-versus-last difference would capture, nor is it robust to isolated extreme values, for which a median would be less sensitive. Including temporal slopes, differences, or lagged values is therefore an important next step, ideally in larger, multi-center cohorts where this can be done without a disproportionate increase in overfitting risk. Furthermore, comparison with PopPK models is not a methodological benchmark since the ML models were trained on a local cohort and PopPK models were developed using external datasets. However, ML models generally outperform PopPK models on their native cohort. The ability of ML approaches to outperform PopPK models using routine data suggests that their performance may improve with larger or more informative datasets. While initial dosing is the focus, future studies should evaluate dosing decisions and serial TDMs to determine the impact of ML-based prediction on antibiotic therapy. A related limitation is that the model used the cumulative vancomycin dose as a single predictor rather than separating loading dose and maintenance rate. Although the dosing records allow this distinction, the bolus dose varied only minimally across patients and carried little additional signal on its own. As the two are decided separately in clinical practice, separating their contributions remains an important next step in cohorts with more heterogeneous bolus dosing. We also note that the initial vancomycin concentration, rather than the AUC recommended by current guidelines, was chosen as the primary outcome, because reliable AUC estimation requires multiple concentrations per dosing interval or a Bayesian estimate on an established population PK model, neither of which was consistently available in this retrospective cohort. Because our patients received a loading dose followed by continuous infusion, the predicted concentration is nonetheless closely related to the guideline target: under continuous infusion, the steady-state 24-hour AUC is proportional to the steady-state concentration. The model is therefore best positioned as an early, pre-TDM estimate of expected exposure that can flag likely under- or over-dosing at the first dose, complementing rather than replacing subsequent AUC-guided monitoring. A follow-up study with more systematically collected measurement time points is planned to extend the approach toward direct AUC prediction. Prospective validation and integration into clinical workflows will be essential prior to implementation. Decision-support tools may require regulatory evaluation since they may be classified as medical devices. Prospective validation in diverse ICU cohorts is an essential next step. Integration into clinical workflows could determine the model’s feasibility and clinical impact, including estimation of concentrations for initial doses. The work demonstrates the potential of routinely collected data for more comprehensive dosing support. Methodologically, larger multicenter datasets will enhance generalizability and identify robust predictors. Incorporating temporal information may improve modelling of pharmacokinetics in sepsis. As these approaches mature, they may support the development of streamlined PDMS-integrated dosing-support systems.

## Conclusion

In conclusion, this study demonstrates that machine learning can reliably predict initial vancomycin serum concentrations in septic ICU patients using routinely available clinical data and can outperform traditional population pharmacokinetic models. In this setting, the elastic net model enabled accurate and clinically interpretable predictions across subtherapeutic, therapeutic, and supratherapeutic concentration ranges, even before TDM results were available. The selected predictors and their quantitative effect sizes reflected both established pharmacokinetic principles and complex pathophysiological alterations characteristic of sepsis, linking patient-specific clinical conditions to measurable changes in drug exposure. Together, these findings illustrate how ML-based approaches can complement pharmacometric modelling and support earlier, more individualized dosing decisions in critical illness.

## Additional file

Supplementary file 1 (pdf 678 KB)

## Data Availability

The datasets analysed during the current study are not publicly available due to patient data confidentiality. All modelling, aggregation, pre-processing and evaluation steps can be tracked in detail in the original R-syntax in the GitHub-Repository https://github.com/mpc-bioinformatics/predict_vancomycin.

## Abbreviations

BMI: Body mass index
CRP: C-reactive protein
GOT: Glutamic oxaloacetic transaminase
ICU: Intensive care unit
MAP: Mean arterial pressure
MAE: Mean absolute error
MIPD: Model-informed precision dosing
ML: Machine learning
NRBC: Nucleated red blood cells
PCT: Procalcitonin
PD: Pharmacodynamic
PK: Pharmacokinetic
PopPK: Population pharmacokinetic
RDW: Red blood cell distribution
RMSE: Root mean squared error
TDM: Therapeutic drug monitoring
VISA: Vancomycin-intermediate *Staphylococcus aureus*
XGBoost: eXtreme Gradient Boosting

## Footnotes

1 In practice, when predicting the initial vancomycin concentration for a newly admitted patient, this information can be entered manually by the prescribing physician, allowing them to inspect the relationship between the intended vancomycin dose and the predicted serum concentration.

2 https://github.com/mpc-bioinformatics/predict_vancomycin
