# Supplementary material for: Development and evaluation of machine learning-based prediction-modelling for initial vancomycin serum concentrations in septic ICU patients using clinical health record data
Source: Intensive Care Med Exp. 2026 Sep 23;14:125. doi: 10.1186/s40635-026-00973-7 (PMC13601424; doi:10.1186/s40635-026-00973-7)
Supplement: Supplementary file 1 [file 40635_2026_973_MOESM1_ESM.pdf]

Supplementary Material

Development and Evaluation of Machine Learning-Based Prediction-Modelling for Initial Vancomycin Serum Concentrations in Septic ICU Patients Using Clinical Health Record Data

Robin Grugel, Britta Westhus, Hartmuth Nowak, Michael Adamzik, Tim Rahmel, Martin Eisenacher

Purpose

The supplementary material provides additional details on the methodological framework and analytical procedures applied in this study. It includes further information on model development, parameter specifications, and implementation aspects of the applied approaches. Moreover, the supplement outlines the computational workflow and supporting analyses used to derive the reported results. These details complement the main text and hopefully enhance the transparency of the study design and methodology.

Methods

Workflow

The flowchart in Figure 1 provides an overview of the two-pronged strategy underlying our modelling approach. The self-tuning model, including all pre-processing and imputation steps, is evaluated using nested cross-validation (15-fold, five-times repeated, both inner and outer loops) and is subsequently applied to the entire dataset to generate an interpretable and practical prediction model for actual concentration forecasts.

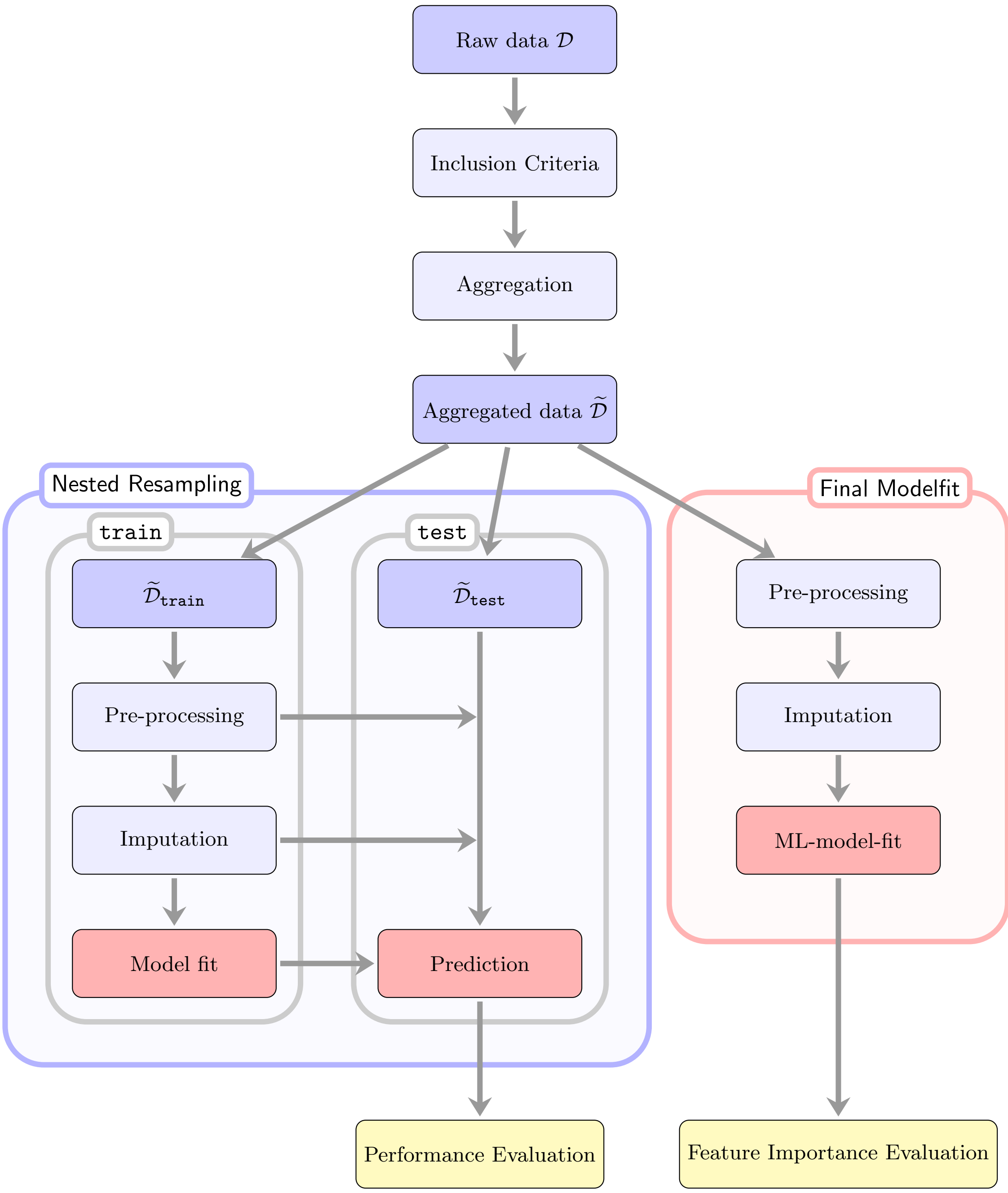

Fig. 1 General modelling workflow chart, from the original raw data to the actual prediction including application of inclusion critera as well as pre-processing steps before and within the resampling procedure for model performance assessments

Modelling and Evaluation Methods

As the true functional form of f is unknown, both linear and non-linear modelling approaches were considered to balance interpretability and flexibility. The concentration $y_i \in \mathbb{R}_{>0}$ of one patient is inherently left-truncated, but will be seen as bounded to the complete real line for sake of simplicity without interfering with the modelling algorithms or results.

Elastic Net

The elastic net as generalization of linear regression introduces penalties on the regression coefficients $\beta = (\beta_1, \ldots, \beta_p)'$ which address different problems. The first issue, numerical instability due to multicollinearity, is typically addressed using ridge regression. It applies an L_2 penalty ($\sum_{j=1}^{p} \beta_j^2$) to shrink coefficients toward zero, without setting any of them exactly to zero, and thus does not create a truly parsimonious model. The second issue, variable selection, can be addressed using the Least Absolute Shrinkage and Selection Operator (LASSO), which applies an L_1 penalty ($\sum_{j=1}^{p} |\beta_j|$) that shrinks some coefficients exactly to zero. The LASSO typically selects one variable from a pair of correlated predictors. To address both issues simultaneously, we employed elastic net regression. This method combines the properties of ridge regression and the LASSO by applying both L_1 and L_2 penalties via

$$p_{\text{EN}} = \sum_{j=1}^{p} \left(\alpha |\beta_j|_1 + \frac{1-\alpha}{2} \beta_j^2 \right)$$

to the regression coefficients, with $\alpha \in [0, 1]$ the mixing parameter controlling the balance between LASSO ($\alpha = 1$) and ridge regression ($\alpha = 0$). The resulting estimate for coefficients can be written as

$$\widehat{\beta} = \arg\min_{\beta_0, \beta} \left\{ \frac{1}{n} \sum_{i=1}^{n} \left(y_i - \mathbf{x}_i' \beta \right)^2 + \lambda \cdot p_{\text{EN}} \right\},$$

where $\lambda \geq 0$ denotes the overall penalty parameter. This formulation allows for both coefficient shrinkage and automatic variable selection, making it especially suitable for data with correlated predictors. The tuning parameters λ and α have to be chosen with a sensible default for parsimonious goal or via cross-validation to optimize predictive performance while maintaining model sparsity. A prediction $\widehat{y}$ for a newly observed individual with predictor set $\widetilde{\mathbf{x}}$ are easily calculated by $\widehat{y} = \widehat{\beta}_0 + \sum_{k=1}^{p} x_j \widehat{\beta}_k$.

Random Forest

A regression tree aims to sequentially partition the predictor space $\mathbf{X}$ for all observations into non-overlapping and distinct regions R_j using cutpoints s_j for a specific predictor $\mathbf{x}_j$ to model the relationship between predictors and outcome. The minimization criterion to select a cutpoint and a predictor in regression trees is the residual sum of squares (RSS)

$$\sum_{j=1}^{J} \sum_{i \in R_j} (y_i - \widehat{y}_{R_j})^2,$$

with $\widehat{y}_{R_j} = \sum_{R_j} \mathbb{1}_{R_j}(\mathbf{X}) \cdot \widehat{y}_{R_j} = \widehat{f}_{\text{tree}}(\mathbf{X})$ being the mean response in the region R_j. A prediction is this average $\widehat{y}_{R_j}$, after a new oberservation $\widetilde{\mathbf{x}}$ was sorted into a region by previously determined set of cutpoints $\mathbf{s}$ for all regions. The Random Forest, first described by Breiman [1], is a prominent algorithm that offers significant advantages over the single regression tree by reducing overfitting and variance through averaging multiple diverse trees, leading to more accurate and stable predictions. A Random Forest regression consists of an ensemble of B regression trees $\{f_b\}_{b=1}^{B}$ [2], where each tree f_b is trained on a bootstrap sample D_b drawn with replacement from the original dataset $D = \{(\mathbf{x}_i, y_i)\}_{i=1}^{N}$, and is therefore part of the bootstrap aggregating

(Bagging) approach. During tree construction, only a random subset of predictor variables is selected. This additional randomization reduces correlation between individual trees and increases ensemble diversity. The prediction for a new observation $\widetilde{\mathbf{x}}$ is obtained by averaging the predictions from all trees via

$$\widehat{y}_{\text{bagg}} = \frac{1}{B}\sum_{b=1}^{B} \widehat{f}_{\text{tree}}^{(b)}(\widetilde{\mathbf{x}}).$$

By aggregating across many weakly correlated trees, Random Forest regression stabilizes predictions and substantially reduces variance compared to a single decision tree, while maintaining low bias. On another note, to address the basic need for parsimonious models, Deng and Runger [3] presented a method to penalise the RSS via multiplying the a regularization factor as a hyperparameter to the splitting criterion. This means that variables with a very low RSS will be picked for a split. The Random Forest has multiple relevant hyperparameters beside the number of predictors considered at each split, the minimum node size or the regularization factor, which have to be determined by a sensible default or tuned in a dedicated resampling procedure.

XGBoost

eXtreme Gradient Boosting (XGBoost), introduced by Chen and Guestrin [4], extends traditional gradient boosting by incorporating additional regularization, efficient handling of sparse data, and parallelized tree construction. Formally, XGBoost seeks to minimize the regularized objective function

$$\mathcal{L}(\phi) = \sum_{i=1}^{n} l(y_i, \widehat{y}_i^{(t-1)} + f_t(\mathbf{x}_i)) + \Omega(f_t),$$

where $l(\cdot)$ denotes a differentiable loss function (e.g., squared error for regression), and f_t represents the tree added at iteration t. The regularization term $\Omega(f_t)$ penalizes model complexity and is defined as

$$\Omega(f_t) = \gamma T + \frac{1}{2}\lambda \sum_{j=1}^{T} w_j^2,$$

with T denoting the number of leaves in the tree and w_j their corresponding leaf weights. The parameters γ and λ control the degree of regularization to prevent overfitting. Predictions are updated sequentially as

$$\widehat{y}_i^{(t)} = \widehat{y}_i^{(t-1)} + \eta f_t(\mathbf{x}_i),$$

where $\eta \in (0, 1]$ is the learning rate controlling the contribution of each tree. The final prediction after M iterations is given by the sum of all base learners. The hyperparameters of the XGBoost model, including the number of boosting rounds M, learning rate η, maximum tree depth, subsampling rate, and regularization parameters γ and λ, were optimized using cross-validation to balance predictive accuracy and generalization.

Hyperparameter Tuning

It should be noted that the number of trees is not considered a hyperparameter for a random forest and should be chosen as large as possible [5], while keeping computational runtime manageable. All selected hyperparameters and their respective ranges are listed in Table 1. Grid search was employed for the elastic net, whereas random search was applied to the random forest and XGBoost due to the time-consuming evaluations of these algorithms and the favorable properties highlighted by Bergstra and Bengio [6]. This strategy ensured adequate coverage of the hyperparameter space while maintaining computational efficiency. For a detailed discussion of advanced hyperparameter optimization strategies, see Bischl et al. [7].

The random search for the optimal hyperparameter configurations of the random forest and XGBoost was performed over 800 potential configurations, reflecting their comparatively high computational cost. The faster-fitting elastic net was evaluated on 2,000 configurations of λ and α."

Table 1 Hyperparameter of the Elastic Net, Random Forest, and XGBoost algorithms with short descriptions and chosen reasonable parameter ranges.

Algorithm	Parameter	Description	Range
Elastic Net[1]	`penalty`	Regularization strength	$[10^{-5}, 10^{2}]$
	`mixture`	Balance between L1 and L2	$(0, 1]$
Random Forest[2]	`mtry`	Number of features per split	$\{1, \ldots, 30\}$
	`min_n`	Minimum samples per leaf	$\{3, \ldots, 7\}$
	`sample.fraction`	Fraction of data used per tree	$[0.5, 1]$
	`replace`	Sampling with replacement	$\{\text{TRUE}, \text{FALSE}\}$
	`regularization.factor`	Regularization strength	$(0, 1]$
XGBoost[3]	`trees`	Number of boosting trees	$\{100, \ldots, 1000\}$
	`sample_size`	Fraction per boosting round	$[0.5, 1]$
	`mtry`	Fraction of features per tree	$[0.1, 0.75]$
	`tree_depth`	Maximum tree depth	$\{2, \ldots, 10\}$
	`learn_rate`	Learning rate	$[2^{-7}, 2^{-1.5}]$
	`alpha`	L1 regularization term	$[2^{-5}, 2^{5}]$
	`lambda`	L2 regularization term	$[2^{-5}, 2^{5}]$
	`loss_reduction`	Minimum loss reduction	$[2^{-10}, 2^{10}]$
	`min_n`	Minimum samples per leaf	$\{1, \ldots, 5\}$

[1]`glmnet` in `parsnip`-modelling interface

[2]`ranger` in `parsnip`-modelling interface

[3]`xgboost` in `parsnip`-modelling interface

Simulation from PopPK-Models

Individual predictions were generated by calculating PopPK-parameters based on the respective patient characteristics (Table 2) and simulating time-concentration curves using the `mrgsolve` package [8] in `R` without incorporating the models random effects. Predicted concentrations were then evaluated at the actual sampling times to compute performance metrics. To achieve accurate predictions, some unit conversions and centralisations individual patient characteristics had to be conducted. The PopPK models used for benchmarking require complete patient-level input for all covariates included in their original specification. Since not all of these covariates were fully recorded for every patient in our cohort, missing values were imputed with plausible population values prior to fitting the benchmarking PopPK models, following the same imputation approach used in the original PopPK model publications. Specifically, missing values were replaced as follows: age was set to 35 years, weight to 70 kg, and height to 170 cm; sex was assumed to be male; dialysis and diabetes status were both assumed to be absent; the SAPS II score at ICU admission was set to 26; and serum creatinine was set to 0.9 mg/dL. This ensures that the PopPK models could be applied consistently across the full cohort, including patients with incomplete covariate data. Listings 2 and 1 provide example code for a one- and two-compartment model, respectively.

Table 2 Formulas to calculate PK parameters based on estimated population effects and individual patient characteristics.

PK-model type	Model	Formula
2-compartment	Goti et al. [9]	$CL = 4.5 \cdot \left(\frac{\texttt{CLcr}_{\texttt{Goti}}}{120}\right)^{0.8} \cdot 0.7^{\texttt{DIALYSIS}}$
		$V_c = 58.4 \cdot \left(\frac{\texttt{BW}}{70}\right) \cdot 0.5^{\texttt{DIALYSIS}}$
		$V_p = 38.4$
		$Q = 6.5$
	Thomson et al. [10]	$CL = 2.99 \cdot (1 + 0.0154 \cdot (\texttt{CLcr} - 66))$
		$V_c = 0.675 \cdot \texttt{BW}$
		$V_p = 0.732 \cdot \texttt{BW}$
		$Q = 2.28$
	Mangin et al. [11]	$CL = 1.91 \cdot 0.66^{(\texttt{SEX})} \cdot \left(\frac{\texttt{BW}}{70}\right)^{0.75} \cdot \left(\frac{\texttt{SAPSII}}{50}\right)^{-0.5} \cdot \left(\frac{88.4 \cdot \texttt{SCR}}{100}\right)^{-0.9}$
		$V_c = 21.9 \cdot \left(\frac{\texttt{BW}}{70}\right)^{0.75}$
		$V_p = 68 \cdot \left(\frac{\texttt{BW}}{70}\right)$
		$Q = 5.71 \cdot 0.3^{\texttt{DIABETES}} \cdot \left(\frac{\texttt{BW}}{70}\right)^{0.75}$
	Okada et al. [12]	$CL = 4.25 \cdot \left(\frac{\texttt{CLcr}}{113}\right)^{0.7}$
		$V_c = 39.2 \cdot \left(\frac{\texttt{BW}}{59.4}\right)^{0.78}$
		$V_p = 56.1$
		$Q = 1.95$
1-compartment	Revilla et al. [13]	$CL = 0.67 \cdot \texttt{CLcr}_{\texttt{Levey}} \cdot 0.06 + \texttt{AGE}^{-0.24}$
		$V = 0.82 \cdot 2.49^{\texttt{A}} \cdot \texttt{BW}$
	Roberts et al. [14]	$CL = 4.58 \cdot \left(\frac{\texttt{CLcr}_{\texttt{BSA}}}{100}\right)$
		$V = 1.53 \cdot \texttt{BW}$
	Adane et al. [15]	$CL = 6.54 \cdot \left(\frac{\texttt{CLcr}_{\texttt{BSA}}}{125}\right)$
		$V = 0.51 \cdot \texttt{BW}$

Notes on PK-Covariates: Estimates for creatinine clearance CLcr (via Cockcroft-Gault formula), $\texttt{CLcr}_{\texttt{Goti}}$ (via Cockcroft-Gault on the serum creatinine value, if SCR < 1 & AGE > 65 then SCR $= 1$, capped at 150 mL/min), $\texttt{CLcr}_{\texttt{Levey}}$ (via Levey formula), $\texttt{CLcr}_{\texttt{BSA}}$ (via Cockcroft-Gault adjusted for BSA using Dubois). BW = body weight (kg), AGE = age (years), SCR = serum creatinine (mg/dL), SAPSII = SAPS II score at ICU admission, SEX (= 1 if female), binary indicators DIALYSIS (= 1 on dialysis), DIABETES (= 1 if diabetic), A (= 1 if SCR > 1). Unit conversions: $88.4 \cdot \texttt{SCR}$ converts µmol/L→mg/dL (Mangin), $\texttt{CLcr}_{\texttt{Levey}} \cdot 0.06$ converts mL/(min · kg)→L/(h · kg) (Revilla).

```
"
$PARAM @annotated
Cli    : 4.5   : Population value for Clearance (L/h)
Vc     : 58.4  : Population value for Central Volume (L)
Vp     : 38.4  : Population value for Peripheral Volume (L)
Q      : 6.5   : Intercompartmental Clearance (L/h)

$CMT
CENT
PERI

$MAIN
double k12 = Q / Vc;
double k21 = Q / Vp;
double k = Cli / Vc;

$ODE
dxdt_CENT = -k*CENT - k12*CENT + k21*PERI;
dxdt_PERI = k12*CENT - k21*PERI;

$TABLE
double CcE = CENT / Vc;
double Cc = CcE;

$CAPTURE
Cc Cli Vc Vp Q
"
```

Listing 1 example of `mrgsolve`-code for the 2-compartment-model of Goti et al. [9]

```
"
  $PARAM @annotated
Cli : 3.833912   : Population value for Clearance (L/h)
Vi : 149.0514 : Population value for Central Volume (L)

$CMT
CENT

$MAIN
double k = Cli / Vi;

$ODE
// Two-compartment model ODEs
dxdt_CENT = -k*CENT;

$TABLE
double CcE = CENT / Vi;
double Cc = CcE;

$CAPTURE
Cc Cli Vi
"
```

Listing 2 example of `mrgsolve`-code for the 1-compartment-model of Revilla et al. [13]

Computational Resources

Listing 3 provides an overview of the utilized R version and the corresponding packages (including their versions), thereby enabling reproducibility of the workflow and the associated processing steps.

```
R version 4.5.2 (2025-10-31)
Platform: x86_64-pc-linux-gnu
Running under: Ubuntu 24.04.4 LTS

Matrix products: default
BLAS:   /usr/lib/x86_64-linux-gnu/openblas-pthread/libblas.so.3
LAPACK: /usr/lib/x86_64-linux-gnu/openblas-pthread/libopenblasp-r0.3.26.so;  LAPACK version 3.12.0

locale:
 [1] LC_CTYPE=en_US.UTF-8       LC_NUMERIC=C               LC_TIME=en_DK.UTF-8
 [4] LC_COLLATE=en_US.UTF-8     LC_MONETARY=en_DK.UTF-8    LC_MESSAGES=en_US.UTF-8
 [7] LC_PAPER=en_DK.UTF-8       LC_NAME=C                  LC_ADDRESS=C
[10] LC_TELEPHONE=C             LC_MEASUREMENT=en_DK.UTF-8 LC_IDENTIFICATION=C

time zone: Europe/Berlin
tzcode source: system (glibc)

attached base packages:
[1] stats     graphics  grDevices utils     datasets  methods   base

other attached packages:
 [1] ggrepel_0.9.6      shapviz_0.10.2     tikzDevice_0.12.6  kableExtra_1.4.0
 [5] tables_0.9.31      RColorBrewer_1.1-3 ggh4x_0.3.1        future_1.67.0
 [9] yardstick_1.3.2    workflowsets_1.1.1 workflows_1.2.0    tune_1.3.0
[13] rsample_1.3.1      recipes_1.3.1      parsnip_1.3.2      modeldata_1.5.1
[17] infer_1.0.9        dials_1.4.1        scales_1.4.0       broom_1.0.9
[21] tidymodels_1.3.0   mrgsolve_1.6.1     DescTools_0.99.60  glmnet_4.1-10
[25] Matrix_1.7-4       ranger_0.17.0      devtools_2.4.5     usethis_3.2.0
[29] openxlsx_4.2.8     vroom_1.6.5        lubridate_1.9.4    forcats_1.0.0
[33] stringr_1.5.1      dplyr_1.1.4        purrr_1.1.0        readr_2.1.5
[37] tidyr_1.3.1        tibble_3.3.0       ggplot2_3.5.2      tidyverse_2.0.0

loaded via a namespace (and not attached):
  [1] jsonlite_2.0.0      rstudioapi_0.17.1   shape_1.4.6.1       magrittr_2.0.3
  [5] rmarkdown_2.29      farver_2.1.2        fs_1.6.6            vctrs_0.6.5
  [9] memoise_2.0.1       htmltools_0.5.8.1   haven_2.5.5         xgboost_1.7.11.1
 [13] cellranger_1.1.0    parallelly_1.45.1   htmlwidgets_1.6.4   rootSolve_1.8.2.4
 [17] cachem_1.1.0        mime_0.13           lifecycle_1.0.4     iterators_1.0.14
 [21] pkgconfig_2.0.3     R6_2.6.1            fastmap_1.2.0       shiny_1.11.1
 [25] digest_0.6.37       Exact_3.3           furrr_0.3.1         pkgload_1.4.0
 [29] textshaping_1.0.1   timechange_0.3.0    httr_1.4.7          compiler_4.5.2
 [33] proxy_0.4-27        remotes_2.5.0       bit64_4.6.0-1       withr_3.0.2
 [37] backports_1.5.0     pkgbuild_1.4.8      MASS_7.3-65         lava_1.8.2
 [41] sessioninfo_1.2.3   gld_2.6.7           tools_4.5.2         filehash_2.4-6
 [45] zip_2.3.3           httpuv_1.6.16       future.apply_1.20.0 nnet_7.3-20
 [49] glue_1.8.0          promises_1.3.3      grid_4.5.2          generics_0.1.4
 [53] gtable_0.3.6        tzdb_0.5.0          class_7.3-23        data.table_1.17.8
 [57] lmom_3.2            hms_1.1.3           xml2_1.5.1          foreach_1.5.2
 [61] pillar_1.11.0       later_1.4.4         splines_4.5.2       lhs_1.2.0
 [65] lattice_0.22-7      survival_3.8-3      bit_4.6.0           tidyselect_1.2.1
 [69] miniUI_0.1.2        knitr_1.50          svglite_2.2.1       xfun_0.53
 [73] expm_1.0-0          hardhat_1.4.2       timeDate_4041.110   stringi_1.8.7
 [77] DiceDesign_1.10     boot_1.3-32         evaluate_1.0.4      codetools_0.2-20
 [81] cli_3.6.5           rpart_4.1.24        systemfonts_1.2.3   xtable_1.8-4
 [85] Rcpp_1.1.0          readxl_1.4.5        globals_0.18.0      parallel_4.5.2
 [89] ellipsis_0.3.2      gower_1.0.2         profvis_0.4.0       urlchecker_1.0.1
 [93] GPfit_1.0-9         listenv_0.9.1       viridisLite_0.4.2   mvtnorm_1.3-3
 [97] ipred_0.9-15        prodlim_2025.04.28  e1071_1.7-16        crayon_1.5.3
[101] rlang_1.1.6
```

Listing 3 R-Version and detailed list of utilized packages

Results

Figure 2 provides an overview of all preprocessed predictors for the 3 ML models, visualized as a correlation matrix to transparently illustrate the pairwise relationships among the selected features. This matrix has been included in the Supplements to document the correlation structure underlying the predictor set. Importantly, no correlations were observed that would be strong enough to raise practical concerns regarding multicollinearity for the ML algorithms used in this study, supporting the suitability of the full predictor set for model training. Figure 3 illustrates the explored hyperparameter space and the corresponding performance, quantified by the mean RMSE, obtained from the final tuning procedure based on 15-fold cross-validation repeated five times on the entire dataset. The selected optimal combination of α and λ is highlighted. Figure 4 presents the regularization path for the selected α value and additionally highlights the final λ value.

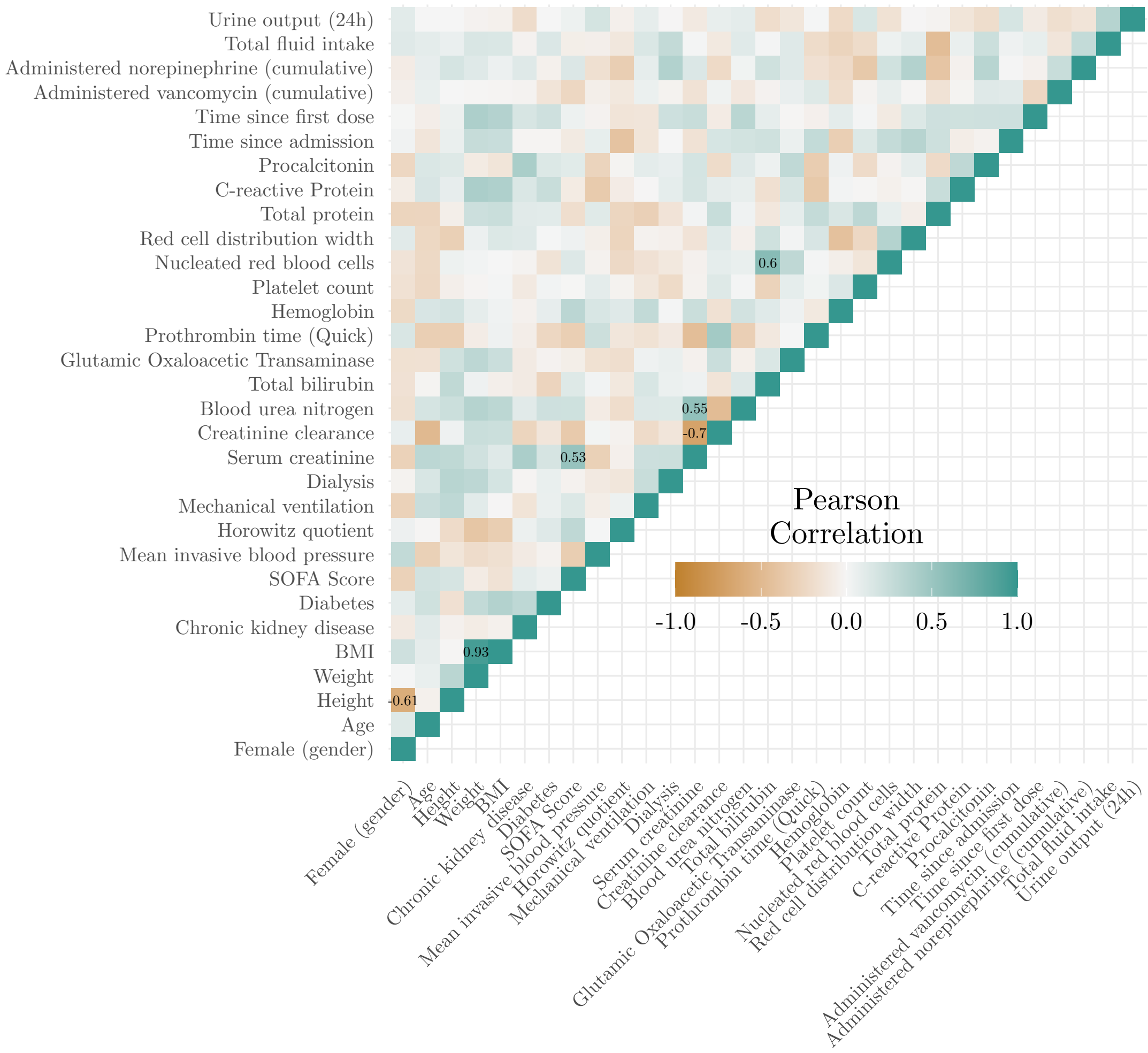

Fig. 2 Pairwise Pearson correlation matrix of the predictors. Predictor pairs with absolute correlation coefficients exceeding 0.5 are explicitly indicated within the matrix.

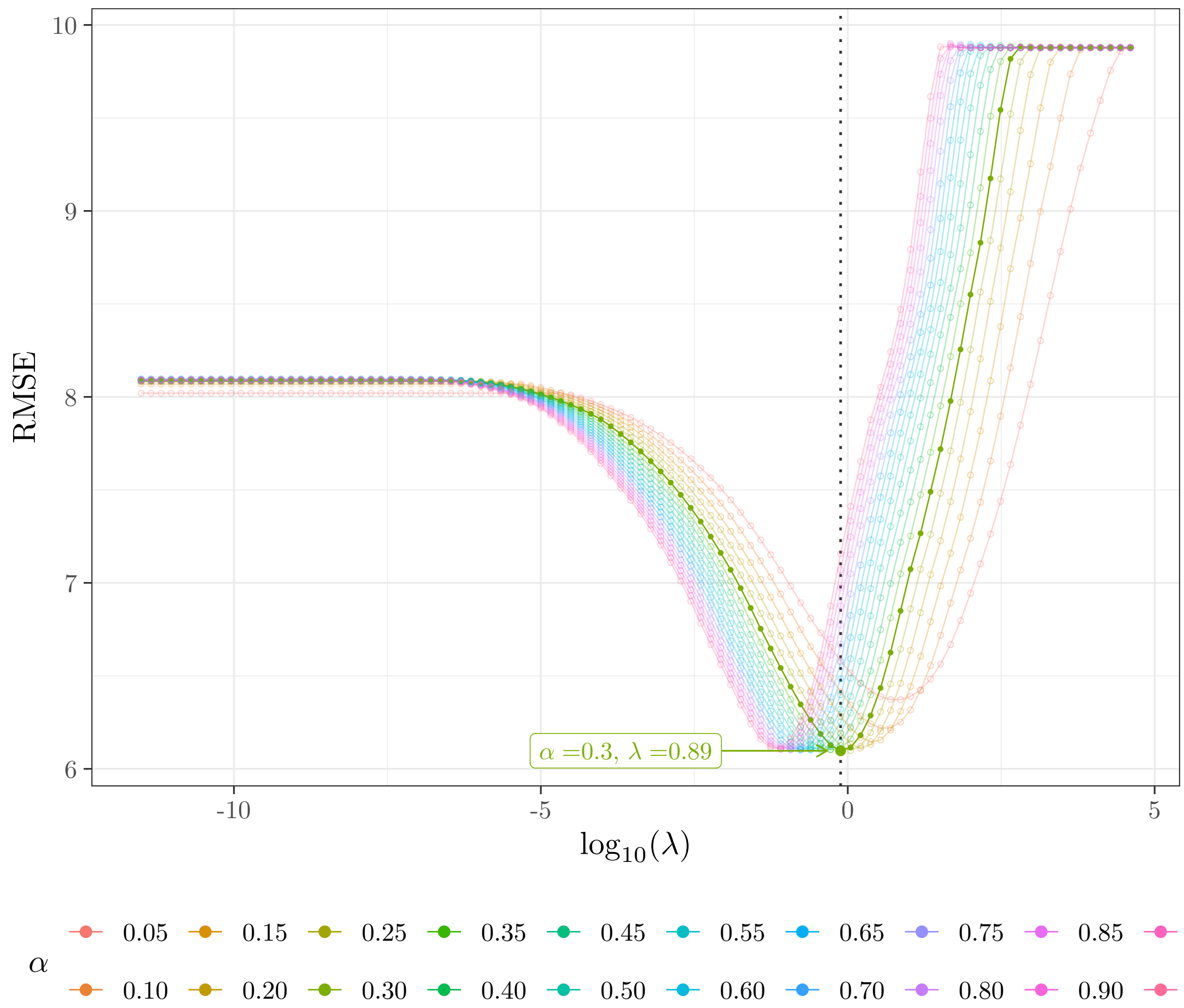

Fig. 3 RMSE of the final tuning on the entire of the data to specify the "optimal" hyperparameter configuration of the Elastic Net regression model.

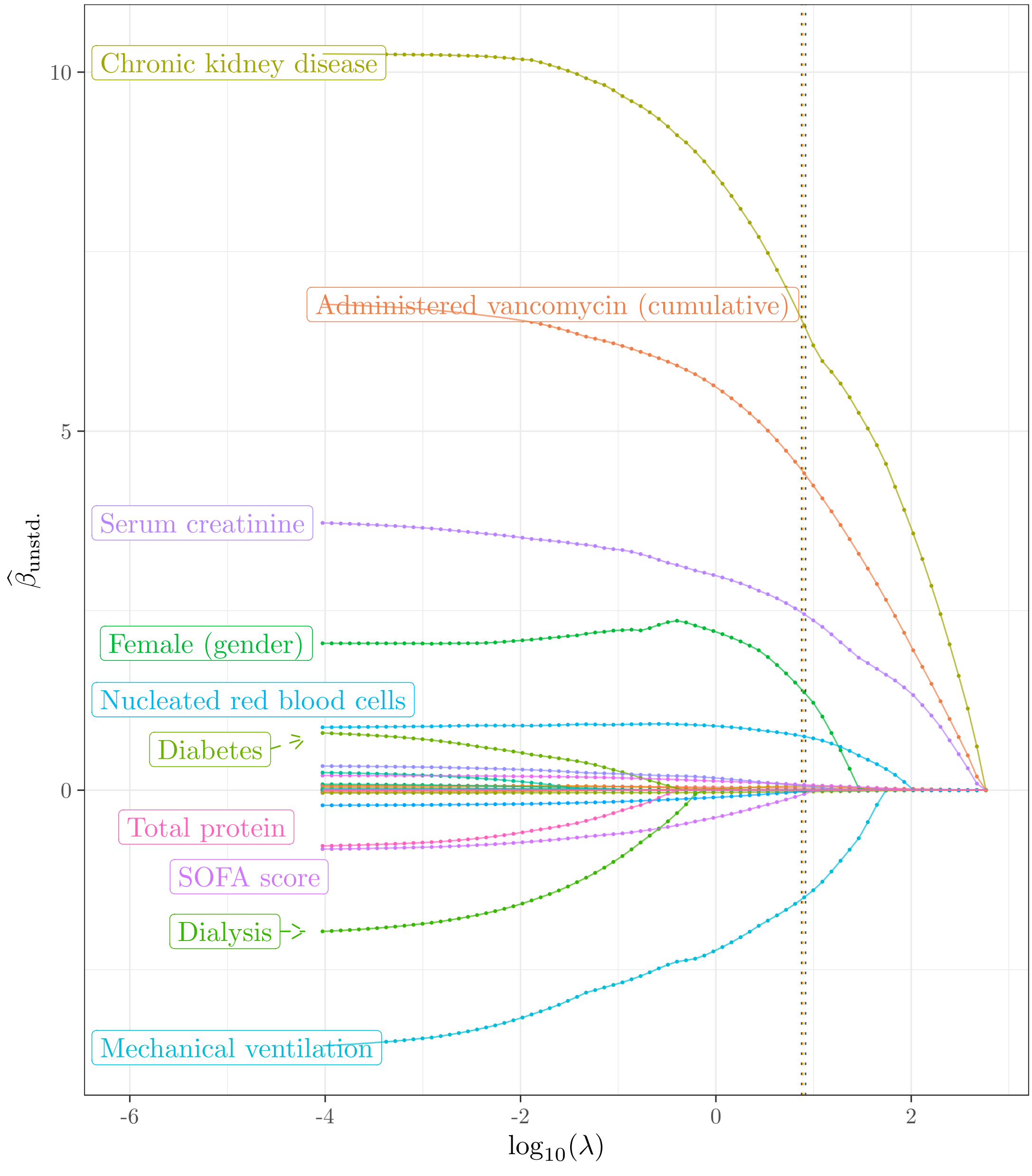

Fig. 4 Regularization path of the final regression fit on the entire of the data with previously tuned hyperparameter configuration; Friedman et al. [16] always provides a complete path of penalization parameter λ.

References

[1] Breiman, L.: Random forests. Machine learning **45**, 5–32 (2001)

[2] Hastie, T., Tibshirani, R., Friedman, J., et al.: The elements of statistical learning. Springer (2009)

[3] Deng, H., Runger, G.: Feature selection via regularized trees. In: The 2012 International Joint Conference on Neural Networks (IJCNN), pp. 1–8 (2012). IEEE

[4] Chen, T., Guestrin, C.: Xgboost: A scalable tree boosting system. In: Proceedings of the 22nd Acm Sigkdd International Conference on Knowledge Discovery and Data Mining, pp. 785–794 (2016)

[5] Probst, P., Boulesteix, A.-L.: To tune or not to tune the number of trees in random forest. Journal of Machine Learning Research **18**(181), 1–18 (2018)

[6] Bergstra, J., Bengio, Y.: Random search for hyper-parameter optimization. The journal of machine learning research **13**(1), 281–305 (2012)

[7] Bischl, B., Binder, M., Lang, M., Pielok, T., Richter, J., Coors, S., Thomas, J., Ullmann, T., Becker, M., Boulesteix, A.-L., *et al.*: Hyperparameter optimization: Foundations, algorithms, best practices, and open challenges. Wiley Interdisciplinary Reviews: Data Mining and Knowledge Discovery **13**(2), 1484 (2023)

[8] Baron, K.T.: Mrgsolve: Simulate from ODE-Based Models. (2025). https://doi.org/10.32614/CRAN.package.mrgsolve . R package version 1.6.1. https://CRAN.R-project.org/package=mrgsolve

[9] Goti, V., Chaturvedula, A., Fossler, M.J., Mok, S., Jacob, J.T.: Hospitalized patients with and without hemodialysis have markedly different vancomycin pharmacokinetics: a population pharmacokinetic model-based analysis. Therapeutic drug monitoring **40**(2), 212–221 (2018)

[10] Thomson, A., Staatz, C., Tobin, C., Gall, M., Lovering, A.: Development and evaluation of vancomycin dosage guidelines designed to achieve new target concentrations. Journal of antimicrobial chemotherapy **63**(5), 1050–1057 (2009)

[11] Mangin, O., Urien, S., Mainardi, J.-L., Fagon, J.-Y., Faisy, C.: Vancomycin pharmacokinetic and pharmacodynamic models for critically ill patients with post-sternotomy mediastinitis. Clinical pharmacokinetics **53**, 849–861 (2014)

[12] Okada, A., Kariya, M., Irie, K., Okada, Y., Hiramoto, N., Hashimoto, H., Kajioka, R., Maruyama, C., Kasai, H., Hamori, M., *et al.*: Population pharmacokinetics of vancomycin in patients undergoing allogeneic hematopoietic stem-cell transplantation. The Journal of Clinical Pharmacology **58**(9), 1140–1149 (2018)

[13] Revilla, N., Martín-Suárez, A., Pérez, M.P., González, F.M., Gatta, M.d.M.: Vancomycin dosing assessment in intensive care unit patients based on a population pharmacokinetic/pharmacodynamic simulation. British journal of clinical pharmacology **70**(2), 201–212 (2010)

[14] Roberts, J.A., Taccone, F.S., Udy, A.A., Vincent, J.-L., Jacobs, F., Lipman, J.: Vancomycin dosing in critically ill patients: robust methods for improved continuous-infusion regimens. Antimicrobial agents and chemotherapy **55**(6), 2704–2709 (2011)

[15] Adane, E.D., Herald, M., Koura, F.: Pharmacokinetics of vancomycin in extremely obese patients with suspected or confirmed staphylococcus aureus infections. Pharmacotherapy: The Journal of Human Pharmacology and Drug Therapy **35**(2), 127–139 (2015)

[16] Friedman, J., Hastie, T., Tibshirani, R.: Regularization paths for generalized linear models via coordinate descent. Journal of Statistical Software **33**(1), 1–22 (2010) https://doi.org/10.18637/jss.v033.i01
